# A role for RASSF1A in tunneling nanotube formation between cells through GEFH1/Rab11 pathway control

**DOI:** 10.1186/s12964-018-0276-4

**Published:** 2018-10-11

**Authors:** Fatéméh Dubois, Bastien Jean-Jacques, Hélène Roberge, Magalie Bénard, Ludovic Galas, Damien Schapman, Nicolas Elie, Didier Goux, Maureen Keller, Elodie Maille, Emmanuel Bergot, Gérard Zalcman, Guénaëlle Levallet

**Affiliations:** 1 Normandie Univ, UNICAEN, CEA, CNRS, ISTCT/CERVOxy group, GIP CYCERON, F-14000 Caen, France; 2 Service d’Anatomie et Cytologie Pathologique, CHU de Caen, F-14033 Caen, France; 3 Normandie Université, Rouen, SFR IRIB, Plateau PRIMACEN, F-76821 Mont-Saint-Aignan, France; 4 Normandie Université, UNICAEN, SFR ICORE, Plateau CMABio3, F-14032 Caen, France; 5 Normandie Université, UNICAEN, UPRES-EA-2608, F-14032 Caen, France; 6 Normandie Université, UNICAEN, UMR 1086 INSERM, F-14032 Caen, France; 7 Service de Pneumologie, CHU de Caen, F-14033 Caen, France; 8 U830 INSERM, “Génétique et Biologie des cancers” Centre de Recherche, Institut Curie, Paris, France; 9 Service d’oncologie thoracique, Hôpital Bichat-Claude Bernard, AP-HP, Université Paris-Diderot, Paris, France; 10 Service D’Anatomie et Cytologie Pathologique, Normandie Univ, UNICAEN, CEA, CNRS, ISTCT/CERVOxy group, CHU de Caen, Avenue de la côte de Nacre, 14032 Caen, France

**Keywords:** Pleural and lung cancer, Tunneling nanotubes, Intercellular communication, RASSF1A, GEF-H1, Rab11

## Abstract

**Background:**

By allowing intercellular communication between cells, tunneling nanotubes (TNTs) could play critical role in cancer progression. If TNT formation is known to require cytoskeleton remodeling, key mechanism controlling their formation remains poorly understood.

**Methods:**

The cells of human bronchial (HBEC-3, A549) or mesothelial (H2452, H28) lines are transfected with different siRNAs (inactive, anti-RASSF1A, anti-GEFH1 and / or anti-Rab11). At 48 h post-transfection, i) the number and length of the nanotubes per cell are quantified, ii) the organelles, previously labeled with specific tracers, exchanged via these structures are monitored in real time between cells cultured in 2D or 3D and in normoxia, hypoxia or in serum deprivation condition.

**Results:**

We report that RASSF1A, a key-regulator of cytoskeleton encoded by a tumor-suppressor gene on 3p chromosome, is involved in TNTs formation in bronchial and pleural cells since controlling proper activity of RhoB guanine nucleotide exchange factor, GEF-H1. Indeed, the GEF-H1 inactivation induced by RASSF1A silencing, leads to Rab11 accumulation and subsequent exosome releasing, which in turn contribute to TNTs formation. Finally, we provide evidence involving TNT formation in bronchial carcinogenesis, by reporting that hypoxia or nutriment privation, two almost universal conditions in human cancers, fail to prevent TNTs induced by the oncogenic RASSF1A loss of expression.

**Conclusions:**

This finding suggests for the first time that loss of RASSF1A expression could be a potential biomarker for TNTs formation, such TNTs facilitating intercellular communication favoring multistep progression of bronchial epithelial cells toward overt malignancy.

**Electronic supplementary material:**

The online version of this article (10.1186/s12964-018-0276-4) contains supplementary material, which is available to authorized users.

## Background

The tumor suppressor gene *RASSF1* (Ras-association domain family isoform) encodes one of the epithelial phenotype guardians [25], RASSF1A, a scaffold protein that maintains cellular homeostasis through control of apoptosis, cell cycle, microtubules stabilization [5, 24, 60] and actin cytoskeleton organization [17, 25]. RASSF1A silencing is a frequent and early event in numerous cancer including lung carcinoma [3, 19] and malignant mesothelioma [22, 74]. In Non-Small Cell Lung Cancer (NSCLC), RASSF1A inactivation is also an independent marker of poor prognosis [19]. RASSF1A depletion underlies tumor initiation and progression [18] since inducing epithelial to mesenchymal transition (EMT) in human bronchial cell lines with a pro-metastatic phenotype sustained by both *i*) guanine nucleotide exchange factor 1 (GEF-H1) inactivation and subsequent RhoB (a putative anti-metastatic small GTPase) inactivation and *ii*) nuclear accumulation of the active form of the Hippo pathway transcriptional cofactor YAP (Yes-associated protein) [25].

Among long and narrow cytoplasmic extensions induced by RASSF1A depletion, some did not seem to contact the substratum [25], which distinguish these structures from mesenchymal morphology or other cell extensions such as filopodia [14]. As previously described in others cell types (see review by Gerdes et al. [27]), these structures reflect a defining characteristic of tunneling nanotubes (TNTs) [62]. It is of note that even TNTs and filopodia share structural similarities, they are different cellular organization that form through different mechanisms [20]. Further, TNTs are divided in two subtypes: Type1 TNT (TNT-1) containing actin and tubulin filaments with a clear cytosolic tunnel that can reach the distances of up to at least 70 μm [28], while Type2 TNT is shorter and contains only actin filaments with unclear cytosolic tunnel. The data in the literature suggest that these two types of TNT could have different functions, as large material (e.g., lysosomes, mitochondria) can only travel between cells via TNT-1 on microtubules [9, 50, 76, 81].

TNT formation has been described as a result of either directed filopodia-like protrusions or from cell dislodgement mechanism [28], although, the molecular basis of their formation still remains under active investigation. To date, actin and its associated proteins such as myosin, small GTPase RalA, filamin, M-Sec and Cdc42 are considered important regulators of TNT formation in various cells, at least in part, through recruitment of exocyst complex to the plasma membrane [12, 29, 33, 64]. In addition, p53 and the PI3K/AKT/mTOR signaling pathways have been demonstrated to be involved in stress-induced TNT formation [80]. Interestingly, EMT was shown to stimulate TNT formation [42]. Finally, the alteration of cell-cell junctions, which occurs after RASSF1A depletion [25, 78] or the disruption of gap junction upon EMT [36], is thought to support the intercellular communication via TNTs [2].

In contrast to gap junctions or micro-vesicles that are most effective in cell communication between relatively close cells, TNTs have been proposed to connect cells located far from each other [41]. TNTs are thus an emerging mode of intercellular communication between cells at both close and distant proximity, already known to facilitate direct transfer of cytosolic molecules and organelles, as well as viral and microbial pathogens from cell to cell [49, 58, 62, 67]. In this respect, TNTs are potential candidates in facilitating the intracellular communication within the heterogeneous tumor microenvironment and have been proposed to be involved in carcinogenesis initiation or later on, in tumor progression [11, 37, 61]. Concordantly, TNTs were identified in different solid resected tumors from patients [76] especially in lung adenocarcinoma and mesothelioma patients [2, 41]. The authors postulate that a higher rate of formation of TNTs is associated with a higher level of local invasion of tumors [2]. Moreover, the transfer of mitochondria via TNT have been shown to enhance chemo-resistance between cancer cell populations [54]. It was also reported that TNT facilitate the exchange of nucleic acid such as mRNA molecules, which could induce or repress the transcription of genes implicated in cancer cell motility or even enhance the transformation of normal neighbor cells [31].

In this regard and given the role of RASSF1A in microtubules stabilization, actin organization and maintenance of epithelial phenotype, we sought to determine how depletion of RASSF1A could affect TNTs formation. We used a various panel of lung epithelial and mesothelial cell lines to study the relation between RASSF1A gene methylation and TNT formation at the initial events leading to bronchial carcinogenesis, as well as in malignant pleural mesothelioma. We employed RASSF1A specific RNAi or a wild-type RASSF1A encoding expression plasmid to assess the consequence of RASSF1A depletion or rescuing on TNTs formation. The role of cellular stressor such as hypoxia or serum starvation has also been explored to recapitulate in vivo cancer microenvironment conditions. Furthermore, we looked for additional key proteins, which are perturbed by loss of RASSF1A and could play a role in TNT formation. These experiments reveal the role of RASSF1A/GEFH1 signaling as a novel signaling module candidate required for TNTs formation, through control of Rab11 accumulation and subsequent exosome release.

## Methods

### Cell culture and treatments

Isogenic HBEC-3 and HBEC-3-KRasV12 bronchial epithelial cell lines were a generous gift of Dr. White (UT Southwestern Medical Center, Dallas, USA) and were cultured in KFSM (Keratinocyte-serum-free medium) complemented with 0.2 ng/ml of human recombinant EGF (Epidermal Growth Factor) and 25 μg/ml of BPE (Bovine Pituitary Extract) supplements (Thermo Fisher Scientific, Rockford, IL). The other cell lines lines were purchased from ATCC. The tumorigenic epithelial cell lines BEAS-2B, BEAS-2B-RasV12, H1975, A549, H1650, H23, and H441 were cultured in DMEM (Dulbecco’s modified essential medium; Gibco), while the mesothelial H2452, H2052, H28 and MSTO-211H cell lines were cultured in RPMI-1640 (Roswell Park Memorial Institute) medium (Invitrogen, Carlsbad, CA) supplemented both with 2 mM of L-glutamine. All the mediums were also complemented with 10% (vol/vol) heat-inactivated fetal bovine serum, 100 U/ml penicillin, 100 μg/ml streptomycin (Gibco). The cultures were incubated at 37 °C in a humidified atmosphere with 5% CO_2_. Where indicated, cells were treated for 24 h before fixation with either paclitaxel (10 nM) to induce microtubule stabilization, nocodazole (10 μM) to induce microtubule depolymerization or blebbistatin (5 μM) to inhibit myosin-II ATPase. To study the influence of metabolic stress, cells were starved in a low-serum medium (0.5% of FBS) for 24 h. Cells were placed in a hypoxic chamber containing 0.1% oxygen, 5% carbon dioxide, and 93% nitrogen for 24 h to determine the influence of environmental stress.

### RNAi, plasmids and transfection procedures

RNAi treatment was performed at 30% confluence, using Lipofectamine RNAiMAX (Invitrogen, Carlsbad, CA) according to the manufacturer’s instructions and analyzed 72 h after treatment. The following RNAi oligonucleotides from Eurogentec® were used: RASSF1A: si1: 5′-GACCUCUGUGGCGACUUCATT-3′ [66] & si2: GAACGUGGACGAGCCUGU [25]; GEFH1: 5′-GAAGGUAGCAGCCGUCUGU-3′ [25]; Rab11a: 5′-UGUCAGACAGA CGCGAAAA-3′ [52]; Rab11b: 5′-GCACCUGACCUAUGAGAAC-3′ [52]; Vimentin: 5′-UCACGAUGACCUUGAAUAA-3′ [57] and non-targeting control RNAi from Dharmacon. Transient transfection with plasmids encoding wild-type RASSF1A (pcDNA3-RASSF1A) and control mimic (Addgene®) were performed using Lipofectamine RNAiMAX (Invitrogen, Carlsbad, CA) following the manufacturer’s instructions and analyzed 24 h after transfection.

### RT-PCR

The mRNA expression was assayed using RT2 ProfilerTM Cell motility PCR Array (Qiagen). Briefly, RNA isolated from HBEC-3 cells transfected with siRASSF1A or siNeg was reverse transcribed and relative gene expression data was obtained using the Human Cell motility PCR Arrays. The expression profile of 84 genes relevant to cell motility as well as five housekeeping genes was assayed. Fold change calculations were done using SABiosciences’ data analysis software which automatically calculates the fold change in gene expression between the treated and control groups.

### Western blot analysis

Protein extraction were performed using a lysis buffer consisting 20 mM Tris (pH 7.0), 50 mM NaCl, 20% glycerol, 5 mM EDTA, and 0.1% TritonX100, 1 mM DTT, 50 mM 2-Mercaptoethanol and 1 μg/ml protease inhibitor (aprotinin, leupeptin, pepstatin A, antipain). Proteins were quantified using Bradford protein assay reagent and stored at − 20 °C. After denaturation (5 min at 95 °C), cell lysates were electrophoresed through SDS polyacrylamide gels, and transferred to nitrocellulose membranes (Hybond-ECL Amersham®). Non-specific sites were blocked for 1 h with Tris-buffered saline containing 0.1% Tween-20 (TBST) and 5% (*w*/*v*) non-fat milk. Membranes were probed overnight with the primary antibody diluted at 1:1000 at 4 °C, and then 1 h at room temperature with the appropriate horseradish peroxidase-linked secondary antibody diluted at 1:2000 in TBST/5%milk. Proteins detected using an enhanced chemiluminescense technique with ECL kit (Promega®).

### Cell labelling and microscopy

#### Labelling of TNT with Alexa 488-WGA

As previously described [9], living cells were labelled with at 5 μg/ml Alexa 488-conjugated WGA (Invitrogen) for 10 min at 37 °C in the CO_2_ incubator. For structural studies, cells were fixed in 4% paraformaldehyde for 5 min and observed in ProLong Gold antifade reagent mounting medium (Invitrogen).

#### Labelling of protein

The primary antibodies used in this study are as follows: anti-RASSF1A (eB114-10H1) from eBioscience, anti-α-tubulin from Sigma Aldrich (St. Louis, MO) and anti-β-actin (8H10D10), anti-cofilin (D3F9), anti-GEF-H1, anti-HIF-1α, and anti-Rab11 from Cell Signaling Technology (Danvers, MA). Briefly, cells were seeded on coverslips at a density of 2 × 10^4^ per well. After treatment, coverslips were washed with phosphate-buffered saline PBS and fixed with 4% paraformaldehyde for 20 min at 37 °C. Cells were permeabilized using ice-cold methanol for 10 min at − 20 °C to preserve microtubules. After extensive washes with PBS, cells were blocked overnight with 5% bovine serum albumin and then stained with primary antibodies and counterstained with appropriate Alexa Fluor 488-, and 555-conjugated secondary antibodies (Thermo Fisher Scientific, Rockford, IL). Coverslips were mounted with DAPI (Santa Cruz Biotechnology), and image captured with high-throughput confocal microscopy (FluoView FV1000, Olympus).

#### Organelle tracker analysis

To determine whether the TNTs allow the transport of mitochondria, transfected cells were divided in two groups. Each group were labeled separately by either Green or Red MitoTracker dyes (Molecular Probes) for 30 min and then washed extensively with PBS before mixing the two populations in the same culture dish. After 1 h of incubation, the cells were either fixed or subjected to the time-lapse imaging. We quantified the number of cells that contain both Mitotracker (yellow color).

To investigate the transfer of either lysosome or endoplasmic reticulum between the cells, Lyso Tracker™ or ER tracker ™ (cell signaling) were added directly into normal growth media for a working concentration of 50 nM or 2 μM respectively according to the manufactures structures and then analyzed immediately by real-time acquisition.

#### Confocal imaging

For living cells, the real-time acquisitions were performed using an inverted microscope (Leica DMi8). The microscopes were enclosed in environmental chambers (Incubator i8) that were maintained at 37 °C with 5% CO_2_ level. The Metamorph 7.8.13.0 software used for image acquisition took a snapshot of four different observation fields every minute for 1 to 3 h. The acquired images were then analyzed with the ImageJ software (version 1.50d).

For fixed cells, images were captured with high-throughput confocal microscopy (FluoView FV1000, Olympus™).

#### gCW STED imaging

Image acquisitions were performed with a 100× oil immersion objective (NA 1.4) through gCW STED imaging (TCS SP5-X; Leica Microsystems) with optimised parameters for Alexa-488 detection. Samples (zoom 8, pixel size = 18.95 nm) were excited with a 488 nm wavelength of a supercontinuum laser. For fixed cells, 20–30% AOTF, conventional scanner (400 Hz, Line Average 1, Accumulation 3, 1024 × 1024) and a step size (0.13 μm) in the xyz mode were used. For living cells, 40–80% AOTF, a resonant scanner (8000 Hz, 1024 × 1024) and a step size (0.13 μm) in the xyz mode were used. Depletion was obtained with a 592 nm laser (70% AOTF). Fluorescence (500–550 nm) was collected with a hybrid detector (Gain 100) in the gated mode and a pinhole for Airy 1. The temperature of the chamber was kept at 37 °C, and cells were provided with constant gas flow (95% O2, 5% CO2) during acquisition with living cells.

### TNTs quantification

After immunolabeling of actin or tubulin filaments, the cells were carefully analyzed for the presence of TNTs, taking into account the criteria previously described in the literature [43]. Those parameters included (a) lack of adhesion of TNT to the substratum of tissue culture plates (b) the width of the extension estimated more than 1 μm and (c) a narrow base at the base of TNT with a triangular aspect called “in arrow”. The number of TNTs for each cell line or after each treatment were counted in 10 randomly chosen fields with 20× objective, and the TNT index was calculated as the average number of TNTs per cell. The TNT lengths were quantified using ImageJ (version 1.50d).

### 3D collagen matrices preparation

Briefly, acid soluble collagen (Nutragen) was brought to physiologic pH on ice to a final concentration of 4 mg/mL. The first layer of the cold collagen solution was then pipetted (100 μL) onto a MatTek dishes (MatTek Co., Ashand, MA) and were then warmed into 37 °C in a cell culture incubator to allow collagen polymerization for at least 30 min. During the time of polymerization, the cells were resuspended in cell culture medium. 2 × 10^5^ cells were added on top of the polymerized collagen layer and placed at 37 °C incubator to allow attachment of the cells to collagen. One hour later, the cells were coated with the second layer of collagen (400 μL) for the establishment of 3D microenvironnement. After 48 h, TNT was imaged using time-lapse microscopy.

### Electron microscopy

#### Transmission

Cells were rinsed in PBS Buffer, fixed with 2.5% glutaraldehyde in phosphate buffer 0.1 M pH 7.4 during 1 h at 4 °C, then rinsed in phosphate buffer 0.1 M pH 7.4 and post-fixed 1 h with 1% osmium tetroxide in phosphate buffer 0.1 M pH 7.4 (at 4 °C protected from light). After washing, cells were dehydrated in progressive bath of ethanol (70–100%), embedded in resin Epon and polymerized 24 h at 60 °C. Ultrathin sections were done and contrasted with uranyl acetate and lead citrate. The cells were observed with transmission electron microscope JEOL 1011 and image were taken with Camera Gatan Orius 200 and digital micrograph software. The number of vesicles was quantified by counting the vesicles present for each treatment condition in 10 fields taken from 10 distinct cells. The diameter of all these vesicles was measured using the digital micrograph software linked to the electron microscope.

#### Scanning

Cells, seeded on coverslips, are fixed with 2.5% glutaraldehyde overnight. The coverslips then undergo three rinses of 10 min with the sodium cacodylate buffer solution pH 7.4 at 0.1 M. The samples are then dehydrated with successive alcohol baths at 70, 95 and 100%, three times 10 min each. A final bath of absolute alcohol will be carried out in the critical point apparatus. A critical point apparatus (Leica CPD 030) is used to replace the alcohol with liquid CO_2_ in the cells. After bypassing the critical point of CO_2_ (31 °C and 73 atm), there is a transition from the liquid state to the gaseous state. This technique preserves the surface structure of the samples. The CO_2_ gas is then gently removed and the samples are brought back to room temperature.

The lamellae are glued to a metal plate covered with conductive tape. The samples are then covered with a thin layer of platinum using a dedicated device (JFC1300 JEOL).

### Exosome vesicles quantification

Exosome vesicles released into the culture medium were assayed following the EXOCET kit (exosome quantitation assay kit) and ExoQuick-TC procedures. Cell media were centrifuged at 3000 × g for 15 min to remove any cell debris. The supernatant (5 ml) were transferred to a sterile container, to which 1 ml of ExoQuick-TC was added. The mixed solution was incubated at 4 °C overnight and then centrifuged at 1500 × *g* for 5 min. The pellet was resuspended in 1 ml of PBS. An aliquot of the suspension (20 μL) was mixed with 80 μL of Exosome Lysis Buffer then was incubated at 37 °C for 5 min to release the exosome proteins, vortexed 15 s and centrifuged 1500 × *g* for 5 min to remove debris. Supernatants were transferred into a 96-well plate well to which are added 50 μl of a mixture 1:1 of the EXOCET reaction buffer reagents A and B before being incubated at room temperature for 20 min. Absorbance at 405 nm was read using a spectrophotometer.

### Statistical analysis

Data are represented as the mean ± SEM of experiments performed independently at least three times. To determine statistical significance, a Student’s unpaired *t* test was applied to all experiments. Statistical significance was set at *p* < 0.05.

## Results

### Characterization of the TNTs structure, formation and intercellular exchange in bronchial or mesothelial cells lines

**Fig. 1:**
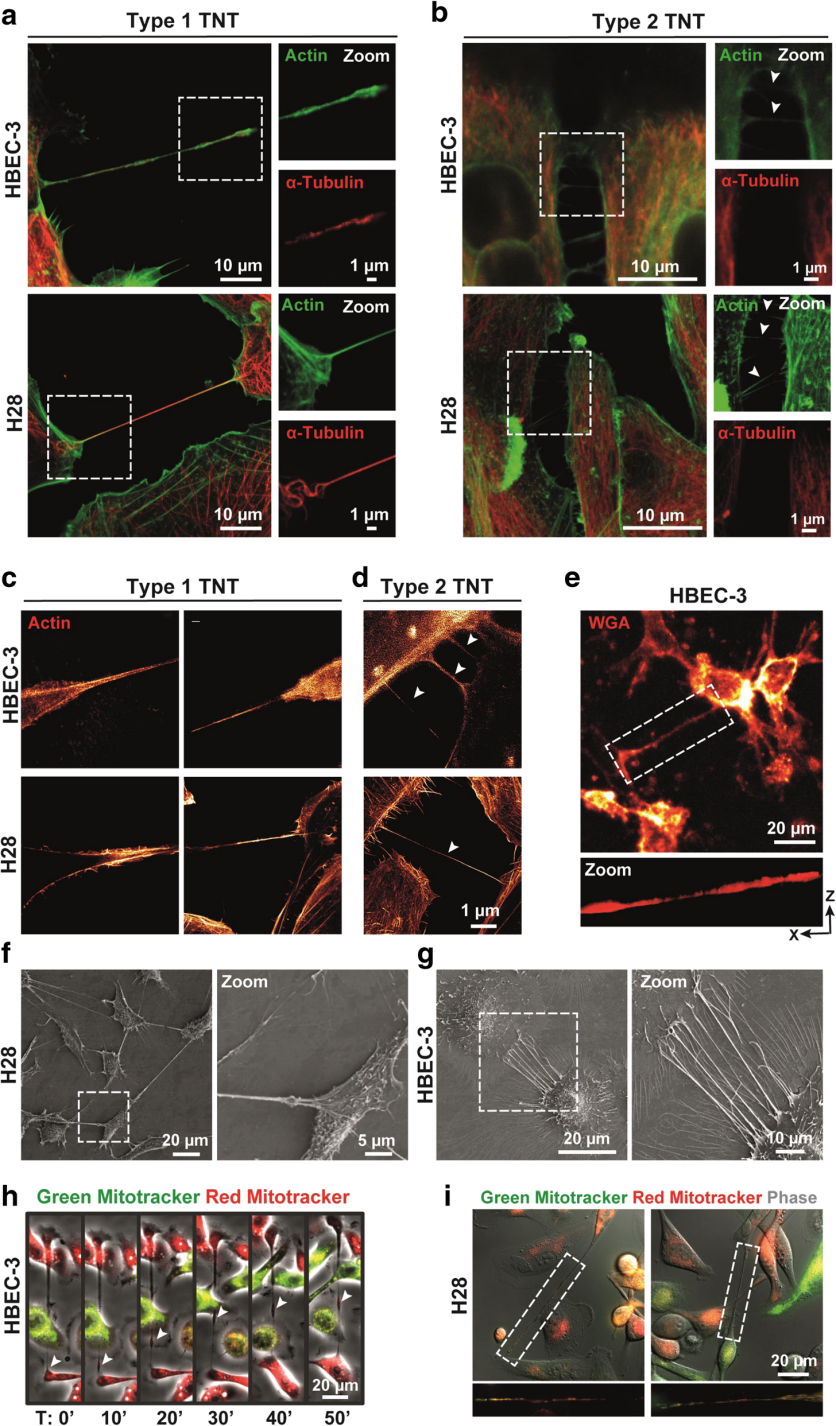
Bronchial as mesothelial cells establish both TNT1 and TNT2 and transfer mitochondria to other cells along TNT1. **a–b** Representative images of cytoskeletal elements presented in either TNT1 or TNT2. HBEC-3 and H28 cells were fixed and labelled with actin (green) and tubulin (red). Boxed regions were used for the zoom of each channel. **c–d** Structural analysis of TNTs subtypes in HBEC-3 and H28 cells through deconvoluted gCW STED nanoscopy. The cells were fixed and stained with Phalloidin for actin filaments. Arrowheads indicate the TNT2. **e** Representative image of TNT between HBEC-3 cells cultivated in 3D collagen matrix along with an optical sections reconstructed in three dimensions. **f**–**g** Representative image of TNT between H28 (**f**) and HBEC-3 (**g**) using scanning electron microscopy. **h**–**i** Montage of MitoTracker dye movement (arrow heads) along TNT in live HBEC-3 (**h**) and H28 (**i**) cells using time-lapse fluorescence video microscopy. Cells were imaged every min for 1 h. See Methods for protocol details


We first wonder whether the nanotubes were observed in various human epithelial bronchial or mesothelial cells lines, supporting their ubiquitous nature. We cultured a panel of immortalized tumorigenic and non-tumorigenic epithelial lung cell lines HBEC-3, HBEC-3-RasV12, BEAS-2B, BEAS-2B-RasV12, lung cancer cells lines H1975, A549, H1650, H23 and H441, as well as mesothelioma cell lines H2452, H2052, H28 and the immortalized MSTO-211H cells, such cell lines being categorizing according their ability to express or not RASSF1A, depending on RASSF1A gene methylation status (Additional file 1: Figure S1A).The TNTs are considered not attached to the substrate as they hover freely in medium and we can observe the bodies of cells and the middle of TNTs in two different optical sections and not with the same focus through microscope [2, 9, 41]. To ensure that it is the case, we either used the time laps (we thus can see that TNTs are even capable of passing above the other attached cells as showed on Additional file 2: Movie S1) or observed TNTs on fixed cells with confocal microscope and not with an epifluorescence microscope to allow discrimination of cells extension touching the substrates from cell bridge (Additional file 3: Movie S2). We report that each cell type exhibited TNTs independently of their genetic background or their tumorigenicity. As illustrated, for one epithelial lung cell line, HBEC-3, and one mesothelial cell line, H28, the observed structures, displayed either the specific characteristics of TNT-1 (type1) with the presence of actin and microtubule structures (Fig. 1a) or filopodia-like TNT-2 (type2), which contains only actin filaments (Fig. 1b) [9]. For more elucidation, we have performed the immunofluorescence experiments with Fascin antibody, a known filopodia marker [77]. We have systematically observed the presence of fascin along actin filaments through thin structure of filopodia (Additional file 1: Figure S1B); however, the results with TNTs remained more variable, where fascin has been detected along TNT in some cells but not others (Additional file 1: Figure S1C). These results are better explained by the two different scenario of TNT formation [1]. Accordingly, we postulate that fascin is present in TNT when they are formed by “actin driven protrusion”, while it is absence when the TNT are issued of “cell dislodgement mechanism”.

Additional file 2: Movie S1. TNTs are cell bridge capable of passing above the other attached HBEC-3 cells. (AVI 561 kb)



Additional file 3: Movie S2. TNT-1 are cell bridge not touching the substrate(here on H2452 cells). (AVI 464 kb)


For further extended demonstration of TNT membrane and morphological structures, we used subsequently STimulated Emission Depletion (STED) nanoscopy, which provides higher lateral resolution. In this regard, the clear cytosolic tunnel, with wider tip on donor cell side and thinner end point on acceptor cell side, was confirmed in TNT1 (Fig. 1c). A cytosolic tunnel was not distinguishable in TNT2 (Fig. 1d), as previously reported [9]. To evaluate TNT formation in a more physiologically relevant microenvironment, the cells were seeded between two layers of type I collagen matrices (see Methods), which has been reported to induce a switch from a 2D to a 3D morphology and to mimic in vivo cellular environment [7]. Consequently, we further confirmed TNT formation in a 3D environment by staining the cells with Alexa 488-wheat germ agglutinin (WGA) (Fig. 1e), a lectin commonly used to stain TNTs [9, 54]. The use of scanning electron microscopy makes it possible to better assess the appearance of TNT formation and its anchoring to the target cell for H28 cells (Fig. 1f) such as HBEC-3 cells (Fig. 1g).

Finally, subsequent studies with time-lapse imaging supported the intercellular communication between cultured HBEC-3 cells via TNT-1 (Additional file 4: Movie S3), as it was also observed by transport of vital mitochondrial dye (MitoTracker) along TNTs in HBEC-3 (Fig. 1h) as H28 (Fig. 1i). The functional activity of TNTs was further reinforced upon transfer of vesicles along TNT-1 using lysosome (LysoTracker, Additional file 1: Figure S1D and Additional file 5: Movie S4) and endoplasmic reticulum (ER-Tracker, Additional file 1: Figure S1E) fluorescent dyes.


Additional file 5: Movie S4. Transfer of vesicles along TNT-1 using lysosome fluorecent dye in HBEC-3. (AVI 288 kb)


### RASSF1A decreases overall TNTs number and length

**Fig. 2:**
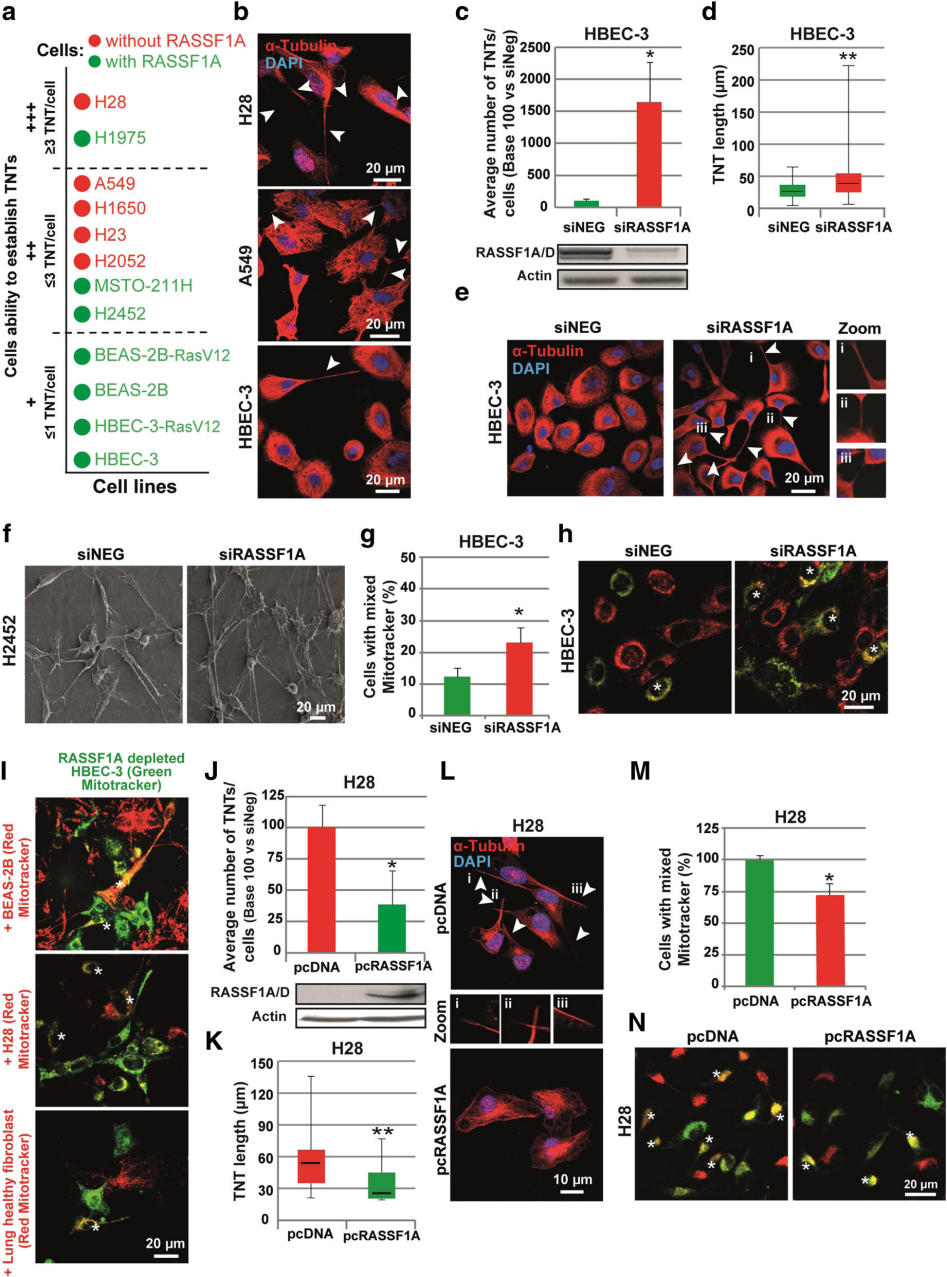
RASSF1A expression decreases overall TNT number and length. (**a**) Quantitative description of the average number of TNTs established for each cell line. TNT’s number was classified as low (+), moderate (++) or high (+++) if there were observed ≤1, 1 ≤ 3 or > 3 TNT per cell respectively. (**b**) Representative images of TNT in HBEC-3, A549 and H28 cell lines fixed and stained with tubulin. (**c**) Quantification of the TNT number and (**d**) length (μm) in HBEC-3 cell line along with (**e**) representative images (ei, eii and eiii are zoom of the corresponding arrowheads). The HBEC-3 cells were treated with control or RASSF1A RNAi as indicated. Western blot showing the efficiency of RASSF1A depletion in HBEC-3 cells 72 h after RNAi treatment. Arrowheads indicate the TNTs. Roman numerals mark the examples of the TNT in the zoomed images. (**f**) Representative image of TNT between H2452 expressing or not (siRASSF1A) RASSF1A using scanning electron microscopy (**g**) Quantification and (**h**) representative images of HBEC-3 cells contained both green and red MitoTracker dyes after RNAi treatment as indicated. (**i**) Representative images of the double labeled MitoTracker dyes in co-culture of RASSF1A-depleted HBEC-3 cells with BEAS-2B, H28 or healthy lung fibroblast cells (**j**) Quantification of the TNT number and (**k**) length (μm) in H28 cell line along with (**l**) representative images (li, lii and liii are zoom of the corresponding arrowheads). The H28 cells were transfected with construct encoding wild-type RASSF1A as indicated. The TNTs are show with arrowheads. Western blot showing the efficiency of RASSF1A transfection in H28 cells 24 h after treatment. (**m**) Quantification and (**n**) representative images of H28 cells contained both green and red MitoTracker dyes after pcDNA transfection as indicated. Values are the mean ± SEM of three independent experiments in almost 200 cells. Statistical significance was calculated and *p* value are indicated by asterisks: *p < 0,05. See Methods for quantification details of each experiments

As RASSF1A was shown to modulate both tubulin- and actin- related structures [16, 25, 40], we then looked for the role of RASSF1A in TNTs formation. We predominantly focused on TNT-1 as they are responsible for the transfer of materials between the cells [50, 81]. We report that all RASSF1A-depleted cell lines (Additional file 6: Table S1) except one, displayed significantly higher number of TNT-1 as compared to the cells with normal basal RASSF1A expression (Fig. 2A-B and Additional file 1: Figure S2A). There was only one exception for the H1975 cell line which exhibited numerous numbers of TNT-1 structures despite a wild type RASSF1A expression (Fig. 2A and Additional file 1: Figure S1A).

To confirm whether influencing RASSF1A expression in vitro could also modulate the occurrence of TNTs formation, RASSF1A knockdown or re-expression was achieved, respectively, in two cell lines with wild type expression of RASSF1A (HBEC-3 and H2452), and two other cell lines (H28 and A549), which RASSF1A epigenetic silencing (Additional file 1: Figure S1A and Additional file 6: Table S1). We observed that extinction of RASSF1A expression in non-tumorigenic HBEC-3 cells, using two different RNA interference [25] (Additional file 1: Figure S2B), increased the average number of TNT-1 per cell (2-fold, *p* < 0.01, Fig. 2C & E and Additional file 1: Figure S2C), as compared to the cells treated with control RNAi. Besides, after RASSF1A depletion, the mean cell-to-cell length of TNT-1 was 1.5 fold higher and could reach up to about 250 μm in length, while control HBEC-3 cells transfected with non-relevant siRNA exhibited TNT-1 reaching not more than 170 μm in length (*p* < 0.01, Fig. 2D-E). For clarity, only the data with one RASSF1A RNAi is shown. The same result was also observed in malignant mesothelioma H2452 cells line after RASSF1A depletion (Additional file 1: Figure S2D, Fig. 2F).

Next, we investigated whether RASSF1A depletion was able to induce functional TNT-1, which allow long distance communication between cells. In this regard, transfected cells were divided in two groups. Each group were labeled separately by either Green or Red MitoTracker dyes (Molecular Probes) before being reseeded together. Time lapse imaging of siNeg or siRASSF1A treated HBEC-3 cells revealed the enhanced transfer of mitochondria after RASSF1A depletion, quantified by cells which contain double labeled MitoTracker dye (Fig. 2G-H). Interestingly, intercellular transfers of cytoplasmic content through TNT-1 occurred similarly between the co-culture of RASSF1A-depleted HBEC-3 cells with different cell line, as shown with the Mitotracker labeled epithelial BEAS-2B cells or mesothelial H28 cells and even healthy lung fibroblast cells (Fig. 2I).

Concordantly, transfection of the plasmid encoding wild type RASSF1A in malignant mesothelioma H28 cell line and lung epithelial A549 (Additional file 1: Figure S2F), decreased significantly not only TNT-1 formation, but also their length and intracellular transfers of mitochondria, compared to the cells transfected with the control plasmid (Fig. 2J-N and Additional file 1: Figure S2E). Taken together, these data provide evidence of the role of RASSF1A in TNTs formation and support the existence of a specific signaling pathway, which leads to an increase of functional TNT-1 after RASSF1A depletion.

### RASSF1A knockdown increases TNTs formation in either hypoxic or serum starved conditions

Environmental or metabolic stresses, such as hypoxia or nutriment starvation, are characteristic of the aggressive tumor microenvironment and have been identified as a hallmark of malignant tumors [63, 85]. The lack of oxygen or nutriments is known to stimulate TNTs formation that serves as an adaptive response to facilitate the connection of the cells at the long distance for the exchange of cytoplasmic materials in order to survive in such hostile conditions [21, 41, 80]. To investigate whether RASSF1A depletion could still enhance TNT-1 occurrence in these conditions, 48 h after transfection, the HBEC-3 cell lines were exposed for another 24 h to either hypoxic environment with 0.1% oxygen or starved in a low-serum medium (0.5% FBS).

**Fig. 3:**
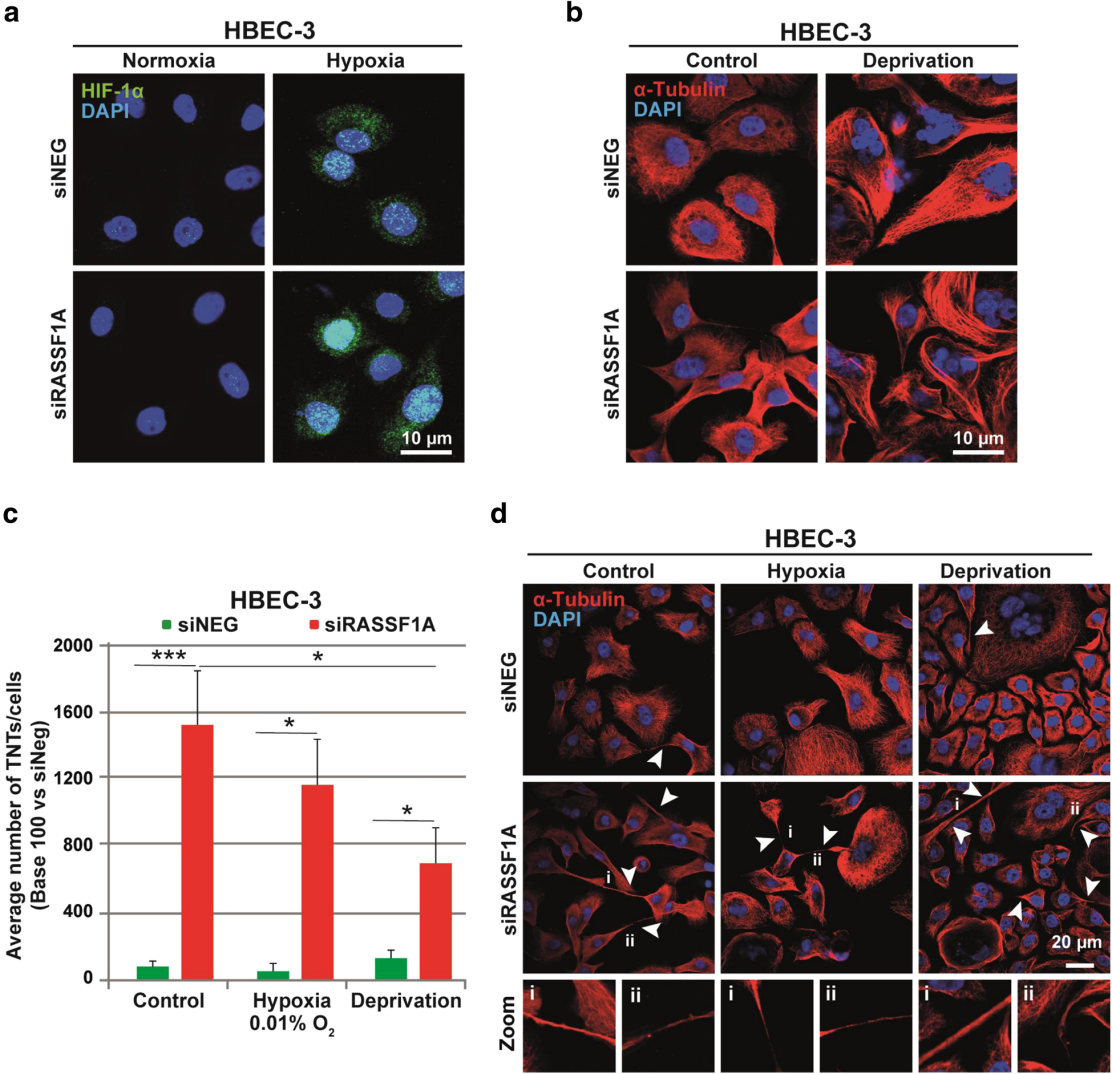
TNTs induced by RASSF1A depletion still occurs in either hypoxic or serum starved conditions. (**a**) Representative images of HIF-1α in HBEC-3 cells incubated for 24 h in normoxic or hypoxic (0.1% O_2_) condition after transfection with either control or RASSF1A RNAi. (**b**) Representative images of the increased number of multi-nucleus HBEC-3 cells, as the result of serum deprivation compared to the cells incubated in normal condition with intact nuclei. (**c**) Quantification of TNT number in control and RASSF1A depleted HBEC-3 cells after incubation in either hypoxic or serum starved conditions and (**d**) Representative images (i and ii are zoom of the corresponding arrowheads for respectively control condition, hypoxia or deprivation treatment). The TNTs are show with arrowheads. Roman numerals mark the examples of the TNT in the zoomed images. Values are the mean ± SEM of three independent experiments in approximately 200 cells. Statistical significance was calculated and *p* value are indicated by asterisks: *p < 0.05, **p < 0,01; *** < 0,001

To confirm the oxygen deprivation in culture, hypoxia inducible factor-1α (HIF-1α) expression was examined by immunofluorescence in the cells exposed to either normoxic or hypoxic conditions, since HIF-1α is known to maintain cellular homeostasis in low oxygen levels and thus can serve as an effective molecular marker of hypoxia [79]. We actually found a nuclear HIF-1α signal in HBEC-3 cell in hypoxic culture condition as compared to the cells cultured in normoxic condition, in which no HIF-1α signal was detected (Fig. 3A). The effect of serum deprivation is also confirmed by the increase of altered-nucleus cells after DAPI staining compared to control cells with intact nuclei (Fig. 3B).

As stated earlier in the cells cultured in standard medium conditions (10% fetal bovine serum and normoxic environment), RASSF1A depletion did increase the number of TNT-1. In either hypoxic environment or in the cells exposed to serum deprivation, RASSF1A depletion still enhanced significantly TNT-1 formation compared to the cells transfected with control RNAi (Fig. 3C-D). These data suggest that RASSF1A promoter methylation, observed at the early pre-invasive stage of cancer development [19], may actually be beneficial for cell survival during environmental or metabolic stresses.

### Vimentin and actomyosin are needed for TNTs formation after RASSF1A depletion

**Fig. 4:**
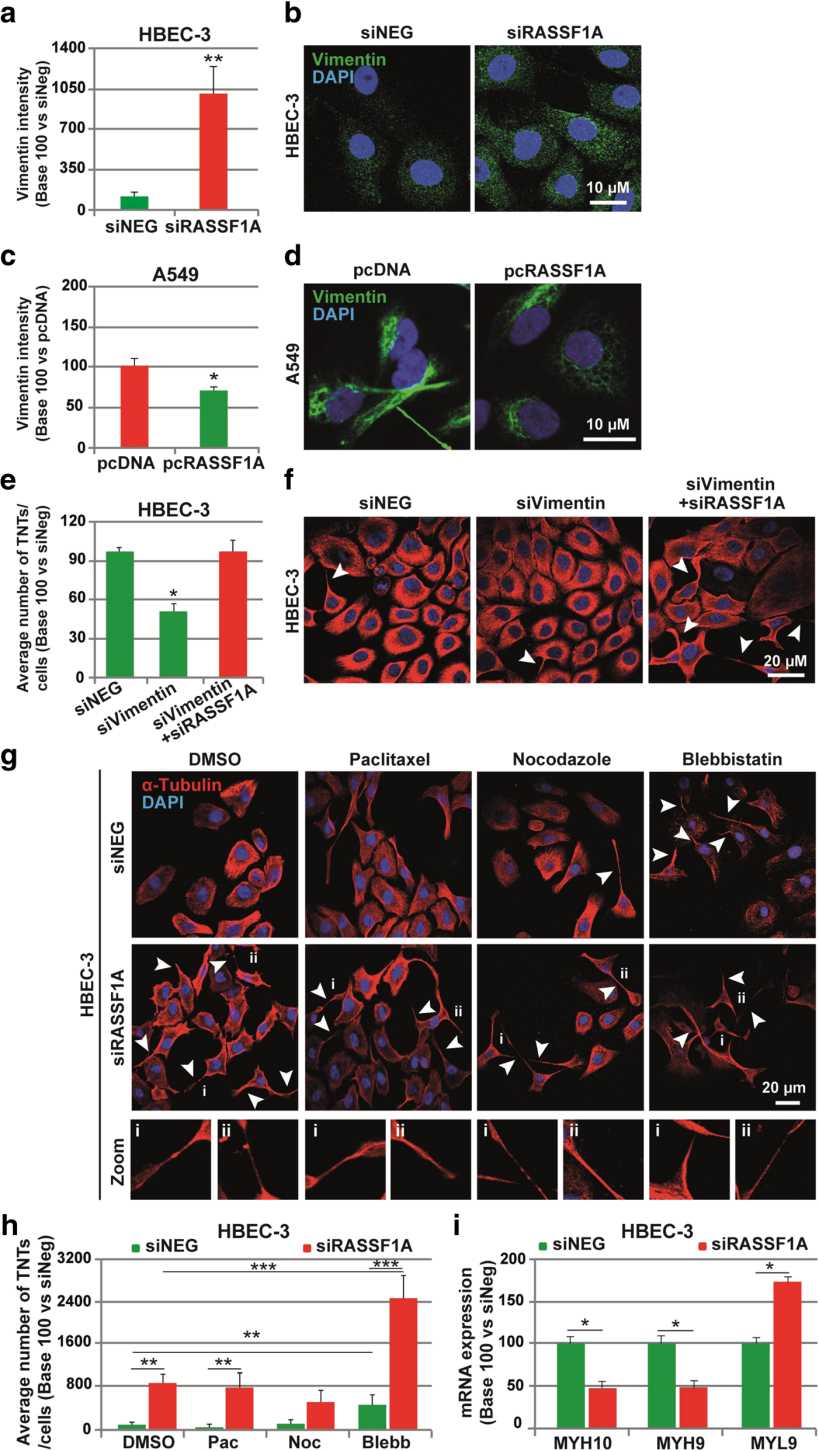
Vimentin and actomysoin are implicated in TNT formation after RASSF1A depletion. (**a**) Quantification and (**b**) representative images of Vimentin expression in HBEC-3 cells transfected with siNEG or siRASSF1A. (**c**) Quantification and (**d**) representative images of Vimentin expression in A549 cells transfected with pcDNA control or RASSF1A. (**e**) Quantification and (**f**) representative images of TNT formation in HBEC-3 cells transfected with either siVimentin alone or in combination with siRASSF1A. (**g**) Representative images (i and ii are zoom of the corresponding arrowheads for respectively DMSO, Paclitaxel, Nocodazole and Blebbistatin treatment) and (**h**) quantification of TNT number in control and RASSF1A depleted HBEC-3 cells after incubation with paclitaxel (10 nM), nocodazole (10 μM) or blebbistatin (5 μM) for 24 h before fixation and staining with α-tubulin. The TNTs are show with arrowheads. Roman numerals mark the examples of the TNT in the zoomed images. (**c**) The mRNA expression was assayed using RT2 ProfilerTM Cell motility PCR Array (Qiagen). β_2_-microglobulin was used as an internal control. Values are the mean ± SEM of three independent experiments in almost 200 cells. Statistical significance was calculated and *p* value are indicated by asterisks: *p < 0.05, **p < 0.01, *** < 0.001

We then sought to determine whether RASSF1A depletion increased the number and length of the functional TNTs by influencing cytoskeletal elements. Indeed, in line with the role of RASSF1A in maintaining epithelial phenotype [25], increase of vimentin expression (marker of mesenchymal cells [44]) in RASSF1A depleted HBEC-3 epithelial cells was concordant with the increase of TNT-1 formation (Fig. 4A-B), while RASSF1A re-expression in A549 cells, significantly reduced both vimentin and TNT-1 number (Fig. 4C-D). Similarly, depletion of vimentin by RNAi, decreased significantly the ability of RASSF1A-knockdown cells to form TNT-1 (Fig. 4E-F). These observations suggest a role for vimentin in TNTs genesis.

We also sought to study the role of other cytoskeletal components, and 48 h after RASSF1A knockdown, HBEC-3 cells were incubated with cytoskeleton disrupting drugs during 24 h, before fixation. Despite the well-known role of RASSF1A in microtubules stabilization [16, 26], stabilization of the microtubule by paclitaxel (10 nM) or their depolymerization with nocodazole (10 μM) did not affect the enhancement of TNT-1 formation induced by RASSF1A knockdown (Fig. 4G-H). In contrast, inhibition of myosin II ATPase with blebbistatin (5 μM) increased not only the TNT-1 formation in control cells, but also showed an additive effect with RASSF1A depletion on enhancement of TNT-1 genesis (Fig. 4G-H). We also find that RASSF1A knockdown reduced the mRNA expression of Myosin 9 and 10 heavy chains mRNAs and increased the expression of Myosin 9 light chain transcript (Fig. 4I). These data confirm the previously identified role of the loss actin bundles in TNTs formation [30, 51], while conversely supporting a new role for RASSF1A as a modulator of TNTs formation, through control of myosin expression and actin filaments contractility.

### Exosomes released by RASS1A depleted cells affect TNT formation

**Fig. 5:**
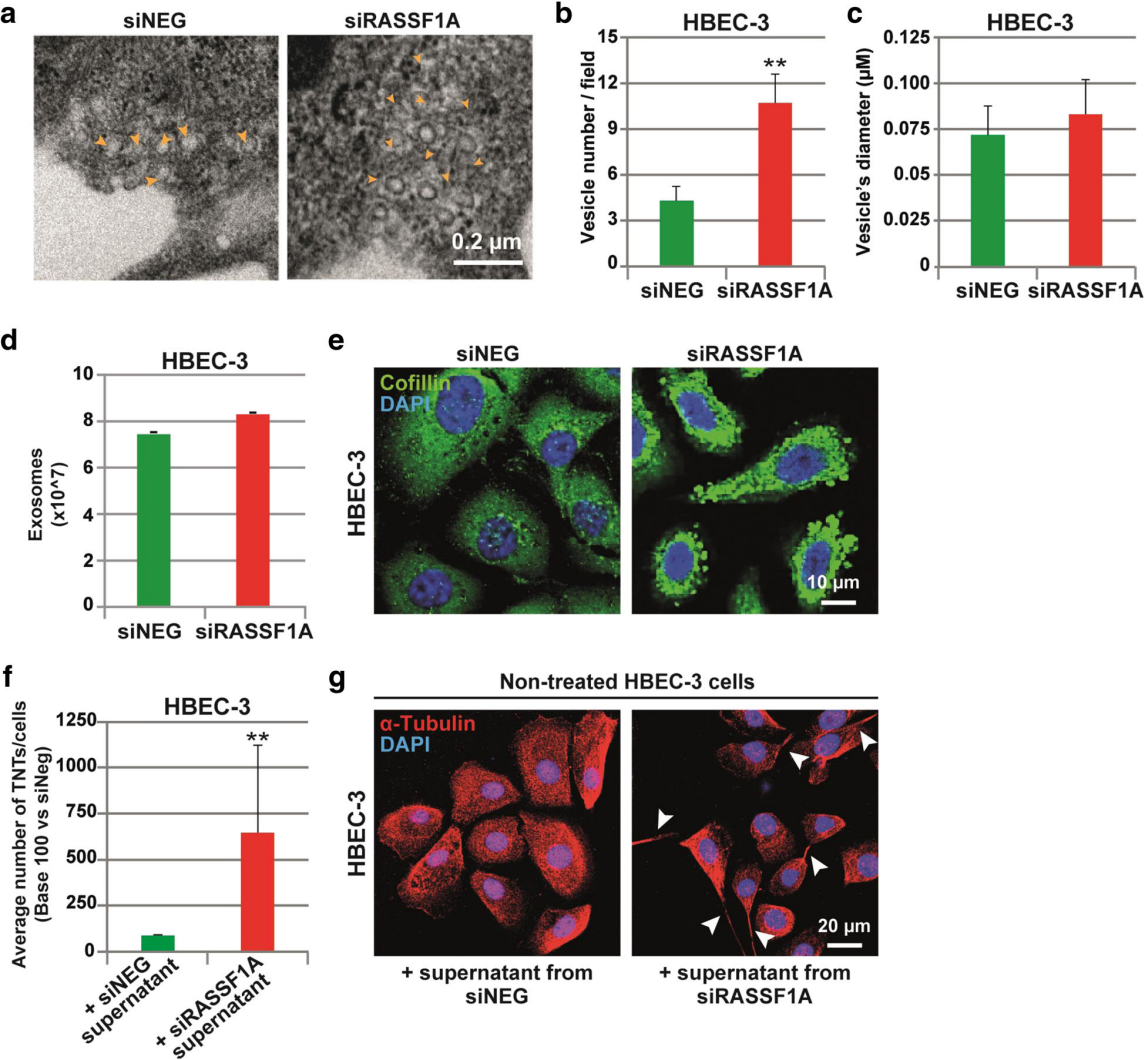
Exosomes released by RASSF1A depleted cells affect TNT formation. **a** Representative images and **b–c** quantification of cytoplasmic vesicles number and diameter (μm) by electron microscopy in HBEC-3 cells after transfection with control or RASSF1A RNAi. The vesicles are show with yellow arrowheads. **d** Quantification of the exosome release in control and RASSF1A knockdown HBEC-3 cells. **e** Representative images of cofilin staining in cells transfected with RNAi as indicated. **f** Quantification and **g** representative images of the TNT formation in non-treated HBEC-3 cells incubated with supernatant obtained from siNEG or siRASSF1A treated cells during 24 h. The TNTs are show with arrowheads. Values are the mean ± SEM of three independent experiments in approximately 200 cells. Statistical significance was calculated and *p* value are indicated by asterisks: **p < 0.01


To gain further insight in the singular role of RASSF1A depletion on TNTs formation, we investigated how TNTs formation was triggered. Recently, vesicles from exosomes were also determined as mediators of TNTs formation in mesothelioma cells [71]. Interestingly, our preliminary observations also revealed the presence of multiple vesicles-like structures in the cytoplasm of RASSF1A-knockdown cells (Additional file 7: Movie S5). A closer look on the images captured by electron microscopy (Fig. 5a), confirmed that RASSF1A depletion did increase the number of vesicles (Fig. 5b), without affecting their diameters (Fig. 5c). To our surprise, the quantification of the exosome released in culture media by RASSF1A knockdown cells did not differ from the control cells (Fig. 5d). Therefore, we postulated that exosome production and content might be influenced by RASSF1A expression. In line with this hypothesis, the immunofluorescence staining of cofilin, a protein implicated in exosome biogenesis and function [73], confirmed a clear increase in cofilin content within exosome structures upon RASSF1A depletion (Fig. 5e).

Additional file 7: Movie S5. Multiple vesicles-like structures are present in the cytoplasm of RASSF1A-knockdown cells. (AVI 936 kb)


To test whether the exosome contents can modulate TNT-1 formation, 48 h after transfection, the medium from siNeg- or siRASSF1A-transfected HBEC-3 cells was collected and transferred to the parental non-treated HBEC-3 cells. Remarkably, 24 h after medium transfer, we noticed a significant increase of TNT-1 formation in HBEC-3 cells incubated with the medium issued from RASSF1A-depleted cells (Fig. 5f–g). This data reveals that the increase of TNT-1 formation after RASSF1A depletion could occur, at least in part, by the secretion-dependent mechanisms and by the release of exocytic vesicles in the extracellular medium.

### TNTs formation induced by RASSF1A silencing simultaneously depends on GEF-H1 inactivation and Rab11 activation

Next, we investigated which potential mechanistic pathway was essential for TNTs formation in RASSF1A-depleted cells. Recently, our group demonstrated that RASSF1A depletion inhibits the anti-metastatic RhoB GTPase via phosphorylation and inactivation of GEF-H1, with potential implications to delay the progression of RASSF1-hypermethylated lung tumors [25]. Interestingly, GEF-H1 was also reported to be required for endocytic and exocytic vesicle trafficking [56]. Therefore, we wondered whether the increase of TNT-1 formation in RASSF1A-depleted cells was actually mediated by GEF-H1 inactivation.

**Fig. 6:**
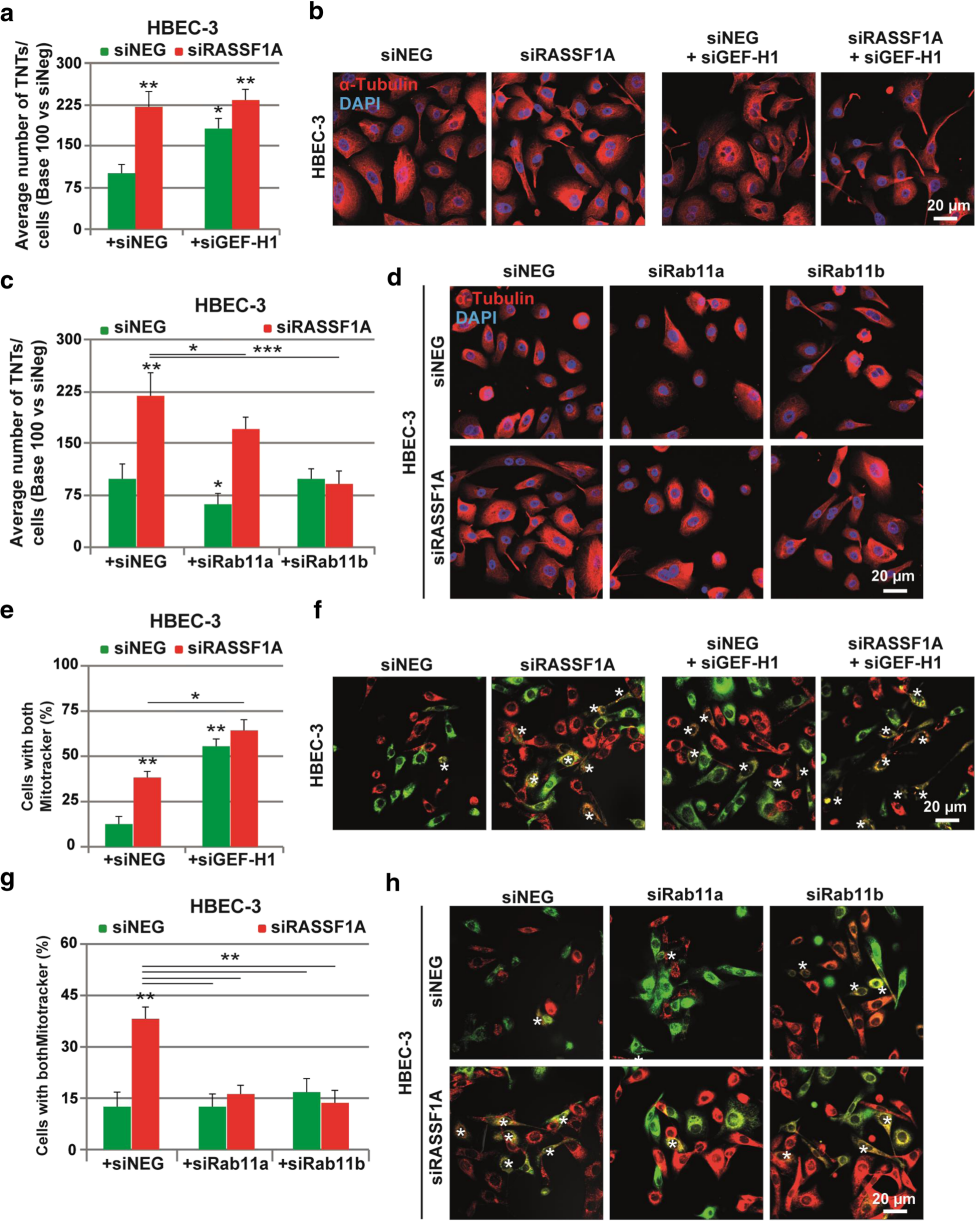
TNTs formation in the absence of RASSF1A is dependent on GEFH1 inactivation and Rab11 activation. **a** Quantification and **b** representative images of the TNT formation in HBEC-3 cells transfected with siNEG or siRASSF1A in combination with siGEFH1. **c** Quantification and **d** representative images of the TNT formation in HBEC-3 cells transfected with siNEG or siRASSF1A in combination with either siRab11a or siRab11b. **e** and **g** Quantification and **f** and **h** representative images of HBEC-3 cells labeled with both green and red MitoTracker dyes after RNAi treatment as indicated. Values are the mean ± SEM of three independent experiments in at least 200 cells. Statistical significance was determined by Student’s t-test and *p* value are indicated by asterisks: *p < 0.05; **p < 0.01; ***p < 0.001. The TNTs are show with arrowheads

In this respect, GEF-H1 inactivation was achieved by siRNA in either HBEC-3 or H2452 cell lines with normal RASSF1A basal expression (Additional file 1: Figure S3A-C). In line with our hypothesis, in both cell types, GEF-H1 depletion significantly elevated the TNT-1 formation in control cells, comparable to the level of TNT-1 number in the cells depleted for RASSF1A (Fig. 6a–b and Additional file 1: Figure S3D-E). Therefore, these data support the idea that the effect of RASSF1A could be mediated by GEF-H1 signaling.

Furthermore, it is known in the literature that loss of GEF-H1 induces the accumulation of Rab11 [55], a small GTPase regulating exocytosis at the plasma membrane [70]. Rab11a and Rab11b are two closely related Rab11 isoforms, which are ubiquitously expressed in most tissue [13]. Previously, Rab11a was also implicated in the TNTs formation [86]. Accordingly, we observed increased expression of Rab11a/b in the absence of RASSF1A (Additional file 1: Figure S3F-G). In this context, we aimed to determine if elevated amount of Rab11 was also involved in the induction of TNTs observed in RASSF1A depleted cells. Hence, by using both Rab11a and Rab11b RNAi in HBEC-3 and H2452 cell lines (Additional file 1: Figure S2H-J), we observed that loss of Rab11a or Rab11b induced a clear and significant reduction in the number of TNT-1 in RASSF1A-knockdown cells, compared with cells treated with siRASSF1A alone (Fig. 6c–d and Additional file 1: Figure S2K-L).

In addition, by quantifying mitochondria transfer between the cells, using MitoTracker, we tested whether altering either GEF-H1 or Rab11 expression, coincided with the modulation of functional TNTs formation, to allow long distance cell-cell communication. Consistent with above data, confocal and time-lapse imaging showed that loss of GEF-H1 in HBEC-3 cell line, led to an increased number of double positive cells, indicative of effective organelle transfer (Fig. 6e–f). Conversely, double knockdown of both RASSF1A and Rab11a, or Rab11b, which decreased significantly the TNT-1 induction, repressed the intercellular mitochondria transfer (Fig. 6g–h). It is of note that the absence of double-labeled mitochondria in individual cell, which did not show any contact via TNTs during time-lapse imaging, argues against the passive dye transfer between the cells. Overall, these results indicate the role of GEF-H1/Rab11 signaling in association with RASSF1A expression, in the control of intercellular communication, by modulating TNTs formation.

## Discussion

We previously showed that the loss of expression of the tumor suppressor gene RASSF1A was a factor of poor prognosis in patient with non-small cell lung cancer [19] since the inactivation of RASSF1A, by altering both the Hippo and Rho signaling pathways, led to the acquisition of a metastatic phenotype by the tumor cells. Here, we hypothesized that the RASSF1A knockdown could also affect intercellular communication via tunneling nanotubes (TNTs), since TNT-1 contains actin and microtubules [9], two elements of the cytoskeleton influenced by RASSF1A cell content [25]. With the present work, we now provide evidence that RASSF1A, by controlling proper GEF-H1/Rab11 activities and cytoskeleton architecture, also prevents TNTs formation in bronchial epithelial or pleural mesothelial cells.

TNTs were first described in vitro in 2004 in the rat pheochromocytoma PC12 cells [62], before being observed in several other cell types (fibroblasts, epithelial cells, immune cells, cardiomyocytes, hippocampal astrocytes, mesenchymal stem cells), as well as in primary cancer cell lines (from ovarian, breast, pancreatic, prostate or colon cancers) [41]. TNTs have then been described ex-vivo in tumor samples from patients with malignant pleural mesothelioma or lung adenocarcinoma [67]. Here, by using a panel of different lung epithelial and mesothelioma cell lines, we confirm that TNTs are ubiquitous cellular structures, since all the cell lines studied displayed cell extensions with the specific characteristics of TNTs (Fig. 1). We also observed that these TNTs enhanced intercellular exchange, time-lapse imaging revealing the presence of organelles such as mitochondria, lysosome or endoplasmic reticulum along TNTs going from one donor to one acceptor cell (Fig. 1 and Additional file 1: Figure S1).

The analysis of the different native lung epithelial and mesothelioma cell lines, as the analysis of lung and mesothelial cells upon RASSF1A silencing, or conversely re-expression pointed out the preferential formation of TNT-1 in RASSF1A-depleted cells, compared to cells with normal RASSF1A expression (Fig. 2). Only H1975 cell line, despite a normal expression of RASSF1A, exhibited numerous numbers of TNTs formation which could be explained by the activating mutation of PI3K on these cells [53], since PI3K was previously implicated in TNTs induction [67, 80].

We demonstrate here that RASSF1A prevents TNT-1 by its control on both actin cytoarchitecture and intermediate filaments components. Indeed, while microtubule and actin are the main building blocks of TNTs genesis and stabilization [27], we provide evidence that TNT-1 induced by RASSF1A silencing in bronchial epithelial or mesothelial cells did not depend on microtubules since TNT-1 were insusceptible to either pharmacologic stabilization of microtubules by paclitaxel or further depolymerization by nocodazole (Fig. 4). While some studies showed the involvement of the microtubules in the formation of TNTs [81], others did not find such a role [39, 59], suggesting that the role of microtubule in TNTs formation, may vary depending on the cells type. Conversely, in line with previous reports, we confirmed the involvements of actin, because the loss of actomyosin filaments network induced by blebbistatin treatment impaired TNTs formation in our cells [27, 30, 32, 51]. RASSF1A depletion enhanced synergistically the effects of blebbistatin to drive extension of TNTs protrusions (Fig. 4). Accordingly, RASSF1A depletion also influenced different myosin’s chains expression. A potential explanation for these results might come from our previous data where RASSF1A depletion was found to influence actin structure through modulating both Rho GTPase and LIMK/Cofilin signaling pathways [25, 68], proteins largely linked to modulation of actomyosin assembly [6, 83]. In addition, we revealed that increase of intermediate filament vimentin, after acquisition of EMT in RASSF1A depleted cells [25], might also contribute to the TNTs formation. Few reports have characterized the presence of intermediate filament such as vimentin along the length of TNTs [2, 75]. Here, we confirmed not only the presence of vimentin along TNTs, but also, we found out that the increase of vimentin expression was concomitant with TNTs formation while vimentin silencing by siRNA decreases TNT-1 formation (Fig. 4). Besides, it is of note that EMT was shown to be a favorable factor to induce TNTs formation [42].

The TNTs induced by the loss of RASSF1A in bronchial epithelial or mesothelial cells are functional and allow the exchange of organelles and in particular of mitochondrial, lysosome or endoplasmic reticulum between cells (Fig. 2). As RASSF1A methylation occurs at the early stage of numerous human cancers and its inactivation is associated with more aggressive tumor phenotype [23, 34], increase of TNT-1 formation in the absence of RASSF1A, could act by stimulating metabolic adaptation of cancer cells at the early stage, and could further participate to the emergence of resistance during drug treatment. Tumor microenvironment is often hypoxic, inflammatory and nutrient-poor during cancer growth. The transfer of different cytoplasmic components including oncogenic genetic materials or nutrients, via TNT-1 has already been suggested to be responsible for the acquisition of functional benefits and phenotypic modifications helping cell survival during environmental or metabolic stresses [2], and even to be responsible for causing and/or maintaining drug resistance [4, 54]. For instance, it has been shown that hypoxia can also induce TNTs-mediated communication [21], and active transfers of mitochondria through TNTs was suggested to rescue aerobic respiration in cells with dysfunctional mitochondria [69]. Importantly, increase of TNT-1 formation in the absence of RASSF1A still occur in either hypoxic or serum starved conditions (Fig. 3). Further, an increased release of exosome was found to be triggered by environmental or metabolic stress [8, 38] as it was observed in a similar way for TNTs formation [21, 41, 80].

Given the ability of RASSF1A to influence LIMK/Cofilin activity [25] and the identified role of cofilin in exosome release [84], another interesting insight was to explore the simultaneous implication of both RASSF1A and exocytosis in TNTs formation. We have observed that the addition of the media issued from RASSF1A-depleted cells was sufficient to enhance the TNT-1 formation in non-treated cells (Fig. 5). Considering this result and in line with previous reports [46, 48, 65], we postulated that the increase of secretion or uptake of exosomes after RASSF1A knockdown could also be introduced as potential chemotactic stimuli acting as paracrine effectors to induce TNT formation. They encapsulate the cytosol of the producing cell containing various effector molecules such as proteins and microRNAs as well as cytoskeleton components including microtubule and actin binding proteins. However, it is still not established whether exosome itself or cytosolic signaling molecules carried within are responsible for the role of exosomes in TNTs formation [35, 45, 72]. Moreover, we cannot completely exclude the possibility that secretion of cytokine and chemokine-based signaling molecules, could act in concert with exosomes to increase TNT formation. In this regard, it has also been shown that TNT stimulate the secretion of prosurvival cytokines [47]. In addition, exosomes are also able to affect cytokines expression profiles [35, 82]. It is of note that characterizing key players of the exosomes or other freely diffusible signals in TNT formation is the main subject of ongoing work in our laboratory.

Mechanistically, we finally demonstrate that RASSF1A prevents TNTs by regulating both the cytoskeleton and exocytosis, since controls proper GEF-H1 and Rab11 activities. Our group has previously demonstrated that RASSF1A knockdown can induce EMT and increase of invasiveness, at least in part, through GEF-H1 inactivation [25]. Besides, it is known that GEF-H1 is a microtubule binding protein, which can also influence the dynamics of the actin filaments by modulating either Rac or Rho activities [10]. Using confocal and time-lapse imaging, we reported that GEF-H1 depletion actually increased both TNTs formation and intercellular transfer between the cells (Fig. 6). Accordingly, RalGPS2, an independent GEF for the Ral GTPase, was also shown to promote the TNTs formation through rearrangement of actin cytoskeleton in bladder cancer [15]. While previously described in Hela cells [55], we confirmed here in bronchial epithelial or pleural mesothelial cells that GEF-H1 depletion did induce Rab11 accumulation (Additional file 1: Figure S3). However, from our results, we suggest that GEF-H1 depletion increase exosome secretion, whereas, Pathak et al., indicate a negative role for GEH-H1 depletion in regulation of exocytosis. Two different hypotheses have been put forward to reconcile these disparate observations. The first, these conflicting data may be explained by context-dependent expression of GEF effectors or their modification. Indeed, the GEF-H1 inactivation induced by RASSF1A silencing, leads only to the decrease of RhoB expression and activity, without affecting RhoA levels [25]. On the contrary, the former study show RhoA activation in response to RalA-Sec5 signaling [55]. Alternatively, differences in cell type or the experimental design in the two studies may be another reason for the opposing results. On the other hands, Rab11 knockdown in RASSF1A-depleted cells, actually decreased the induction of TNTs and subsequent organelle transfer. In line with our results, a recent study demonstrated that downregulation of Rab11 in Schwann cells decreased the formation of functional TNTs and vesicle transfer between the cells [86]. Thus, decrease of GEF-H1 activity, in addition to Rab11 accumulation after RASSF1A knockdown, induces the increase of TNTs formation, possibly by altering exocytosis. In agreement with the role of exocytosis in TNTs formation reported here and by others [46, 65], it is interesting to note that accumulation of Rab11 induced by GEF-H1 depletion [55, 56] was previously shown to regulates exocytosis at the plasma membrane [70].

**Fig. 7:**
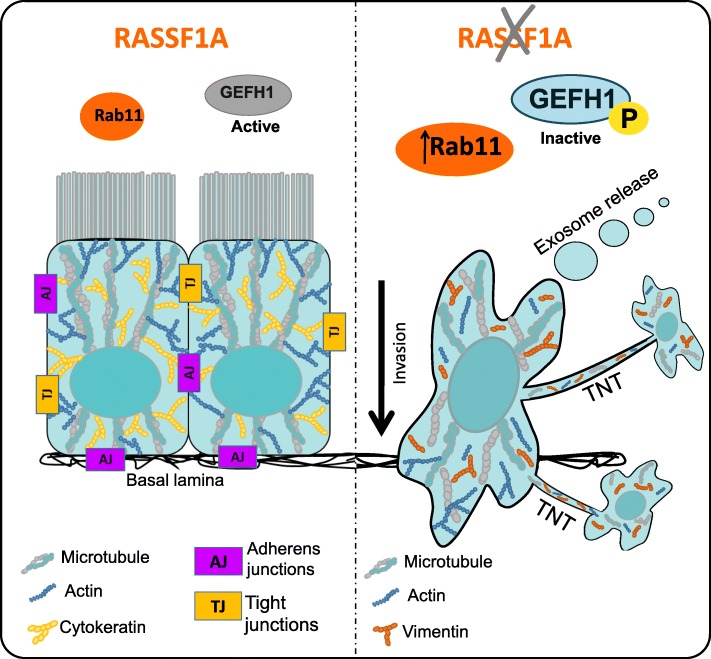
RASSF1A prevents tunneling nanotube formation between cells through GEFH1/Rab11 pathway control

Taken together, we provide evidence here that RASSF1A depletion increased the number and length of functional TNTs. It occurred mechanistically because of *i*) increase of vimentin expression upon the induction of EMT *ii*) loss of actomyosin network through modulating both Rho GTPase and LIMK/Cofilin signaling pathways and *iii*) control of exosome release possibly by inducing GEF-H1 inactivation and RAB11 upregulation (Fig. 7).

## Conclusions

To our knowledge, this study represents the first evidence for a role of RASSF1A in TNTs formation. Thus, we postulate that RASSF1A methylation within the complex and heterogeneous tumor microenvironment would allow the cancer cells to transmit cytoplasmic components (proteins and genetic material) over a long-range distance through TNTs to neighbor cells. Accordingly, targeting TNTs formation appears to consist of a promising therapeutic strategy for preventing development of chemotherapy resistance cancer cells. However, further in-depth preclinical and clinical studies are warranted to investigate the physiologic impact of TNTs within the tumor-stromal matrix and the role of RASSF1A alteration in such phenomenon.

## Additional files

Additional file 1: **Figure S1.** (A) RASSF1A expression in cell lines used in this work by RT-PCR. (B-C) Representative fascin co-staining along actin in both filopodia and TNTs. (D-E)Representative images of (D) Lysosome(LysoTracker) and (E) endoplasmic reticulum (ER-Tracker) along TNT in HBEC-3 cells. **Figure S2**. RASSF1A expression modulates overall TNT number. (A) Representative images of TNT-1 in the cell lines, as indicated (B) immunofluorescence quantification and images indicating a reduction of RASSF1A expression after knockdown (C) Representative image of TNT-1 after RASSF1A depletion by siRASSF1A(2). (D) Quantification of the TNT number along with representative images of siNeg or siRASSF1A transfected H2452 cells. (E) Quantification of the TNT number in A549 cell line along with representative images. The H28 cells were transfected with construct encoding wild-type RASSF1A. Arrowheads indicate the TNTs. Roman numerals mark the examples of the TNT in the zoomed images. (F) Representative image of RASSF1A and actin immunostaining showing the efficiency of pcRASSF1A transfection in RASSF1A-null H28 and A549 cells. Statistical significance was determined by Student’s ttest, *p* value are indicated by asterisks (*p < 0.05). **Figure S3**. TNTs formation induced by RASSF1A loss depends on GEFH1 inactivation and Rab11 activation. (A-B) Immunofluorescence and (C) RT-PCR images showing the efficiency of GEFH1 depletion (D) Quantification and (E) representative images of the TNT formation in H2452 cells transfected with siNEG or siRASSF1A in combination with siGEFH1. (F-G) Immunofluorescence images showing the increase of Rab11 expression after RASSF1A depletion. (H) RT-PCR and (I-J) Immunofluorescence images showing the efficiency of Rab11 depletion in cells 72 h after RNAi treatment. (K) Quantification and (L) representative images of the TNT formation in H2452 cells transfected with siNEG or siRASSF1A in combination with either siRab11a or siRab11b. Values are the mean ± SEM (*n*≥3). Statistical significance was determined by Student’s t-test, *p* value are indicated by asterisks (*p < 0.05;**p < 0.01;***p < 0.001). Arrowheads show TNTs. (PDF 3664 kb)

Additional file 4: **Movie S3.** Intercellular communication between cultured HBEC-3 cells via TNT-1. (AVI 768 kb)

Additional file 6: **Table S1.** Characteristics of the cell lines used in the study. (DOCX 20 kb)

## Abbreviations

EMT: Epithelial-mesenchymal transition
GEF-H1: Guanine nucleotide exchange factor 1
NSCLC: Non-Small Cell Lung Cancer
RASSF1: Ras-association domain family isoform
TNTs: Tunneling nanotubes
