# Supplementary material for: A role for RASSF1A in tunneling nanotube formation between cells through GEFH1/Rab11 pathway control
Source: Cell Commun Signal. 2018 Oct 11;16:66. doi: 10.1186/s12964-018-0276-4 (PMC6180646; doi:10.1186/s12964-018-0276-4)
Supplement: Supplementary file 5 — Figure S1. (A) RASSF1A expression in cell lines used in this work by RT-PCR. (B-C) Representative fascin co-staining along actin in both filopodia and TNTs. (D-E)Representative images of (D) Lysosome(LysoTracker) and (E) endoplasmic reticulum (ER-Tracker) along TNT in HBEC-3 cells. Figure S2. RASSF1A expression modulates overall TNT number. (A) Representative images of TNT-1 in the cell lines, as indicated (B) immunofluorescence quantification and images indicating a reduction of RASSF1A expression after knockdown (C) Representative image of TNT-1 after RASSF1A depletion by siRASSF1A(2). (D) Quantification of the TNT number along with representative images of siNeg or siRASSF1A transfected H2452 cells. (E) Quantification of the TNT number in A549 cell line along with representative images. The H28 cells were transfected with construct encoding wild-type RASSF1A. Arrowheads indicate the TNTs. Roman numerals mark the examples of the TNT in the zoomed images. (F) Representative image of RASSF1A and actin immunostaining showing the efficiency of pcRASSF1A transfection in RASSF1A-null H28 and A549 cells. Statistical significance was determined by Student’s ttest, p value are indicated by asterisks (*p < 0.05). Figure S3. TNTs formation induced by RASSF1A loss depends on GEFH1 inactivation and Rab11 activation. (A-B) Immunofluorescence and (C) RT-PCR images showing the efficiency of GEFH1 depletion (D) Quantification and (E) representative images of the TNT formation in H2452 cells transfected with siNEG or siRASSF1A in combination with siGEFH1. (F-G) Immunofluorescence images showing the increase of Rab11 expression after RASSF1A depletion. (H) RT-PCR and (I-J) Immunofluorescence images showing the efficiency of Rab11 depletion in cells 72 h after RNAi treatment. (K) Quantification and (L) representative images of the TNT formation in H2452 cells transfected with siNEG or siRASSF1A in combination with either siRab11a or siRab11b. Values are the mean ± SEM (n≥3). Statisti [file 12964_2018_276_MOESM1_ESM.pdf]

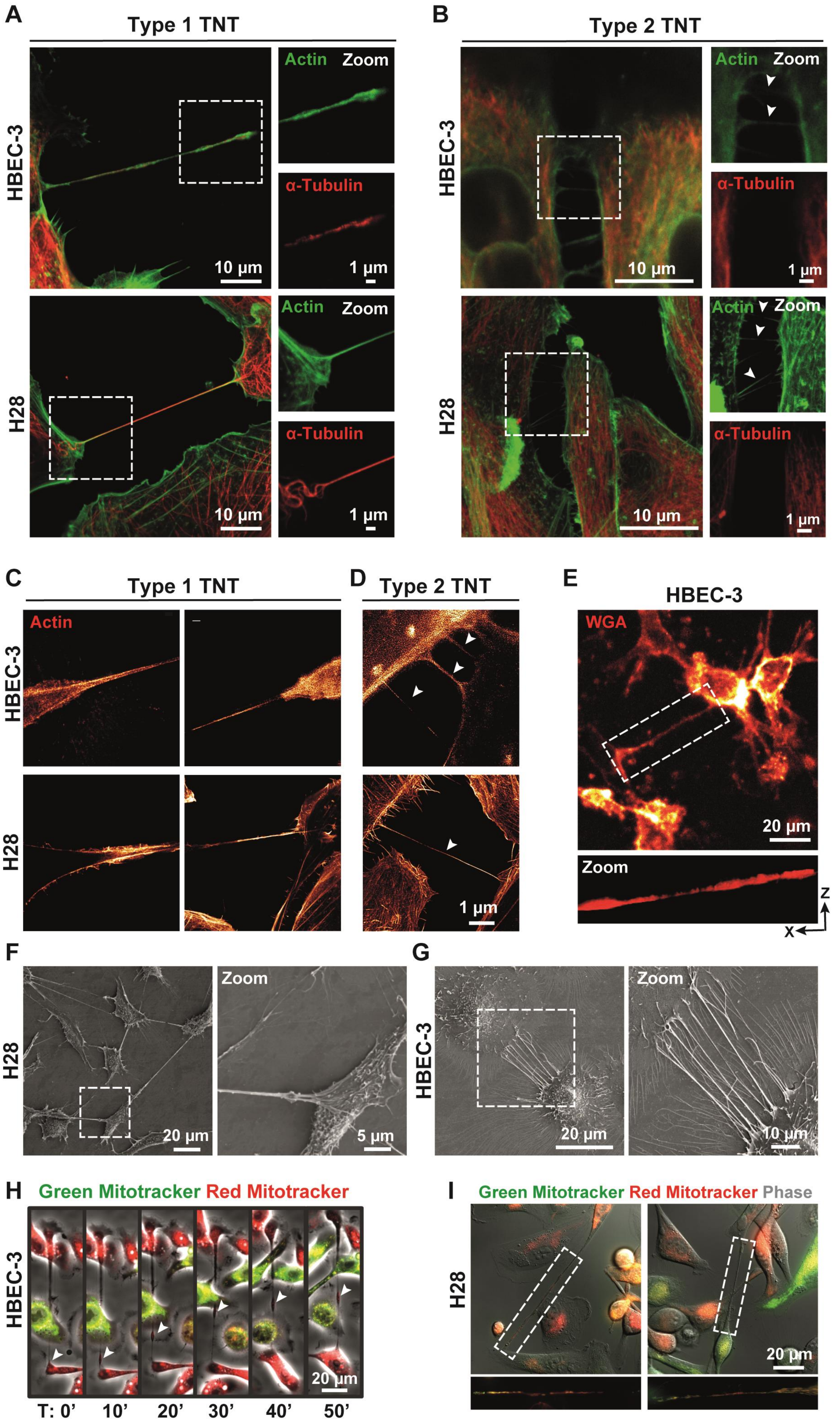

Figure 1: Characterization of the TNTs formation, structure and exchange.

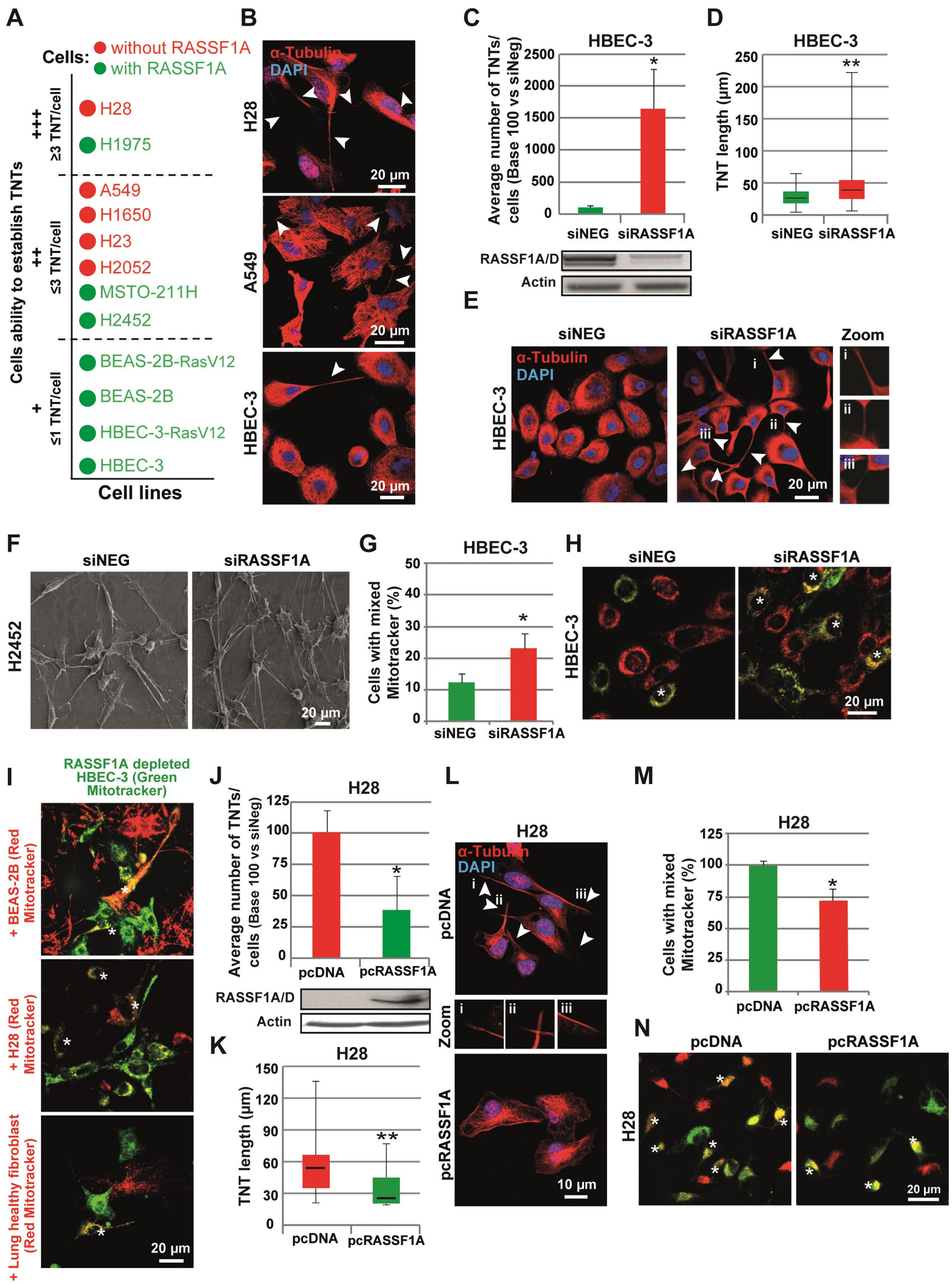

Figure 2: RASSF1A expression modulates overall TNT number.

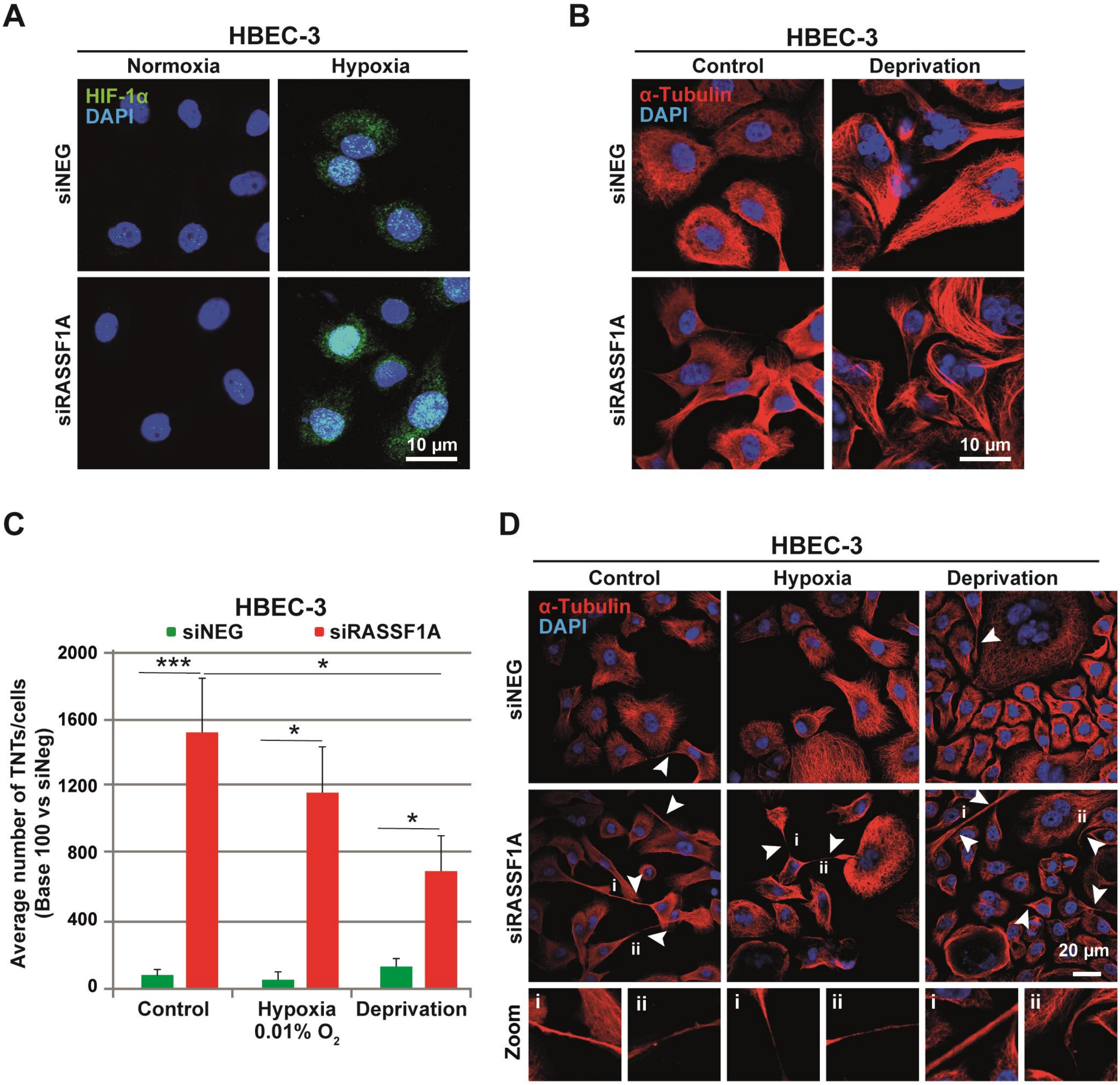

Figure 3: Increase of TNTs formation in the absence of RASSF1A still occur in either hypoxic or serum starved conditions.

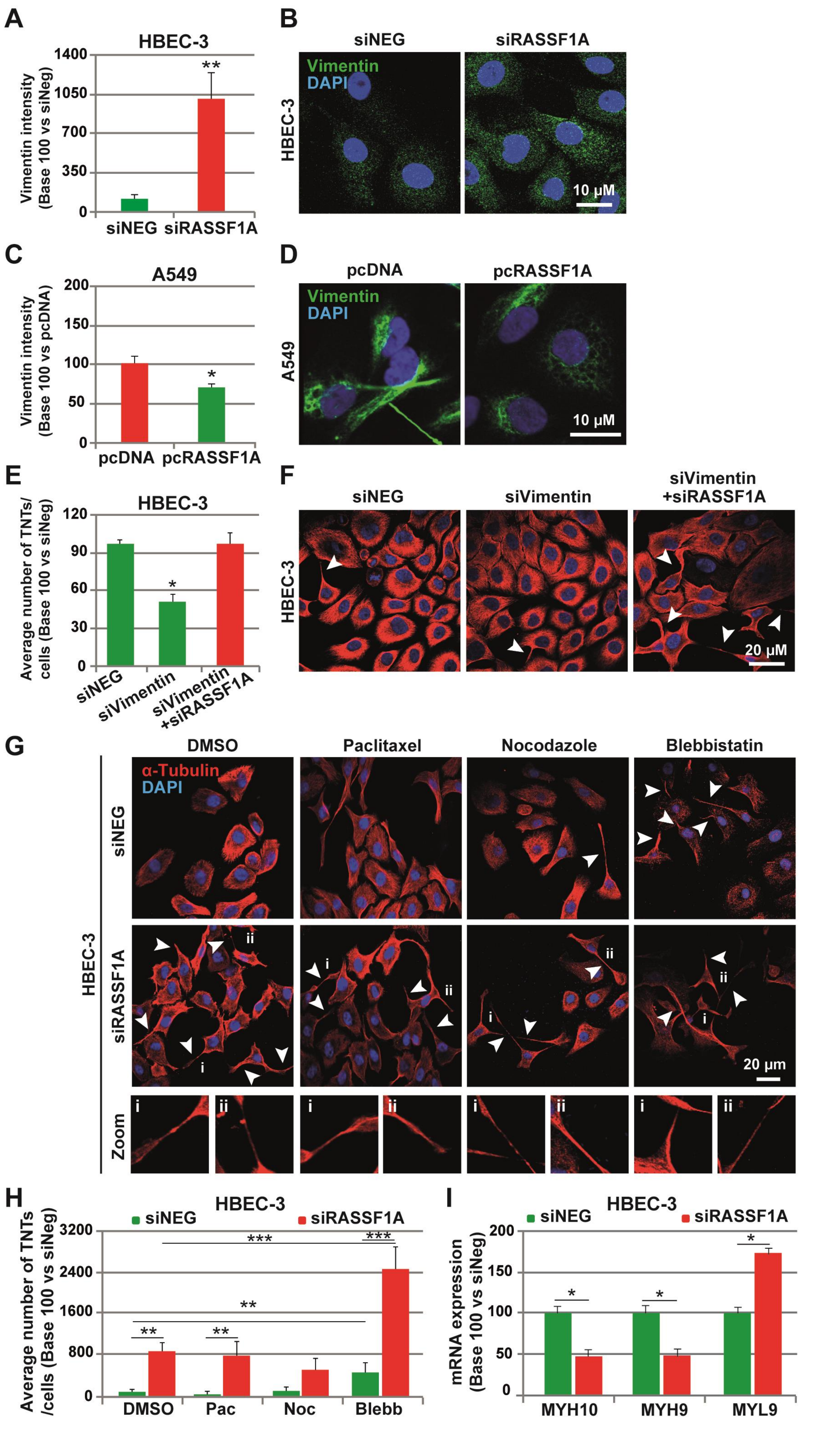

Figure 4: Vimentin and actomyosin are implicated in TNTs formation after RASSF1A depletion.

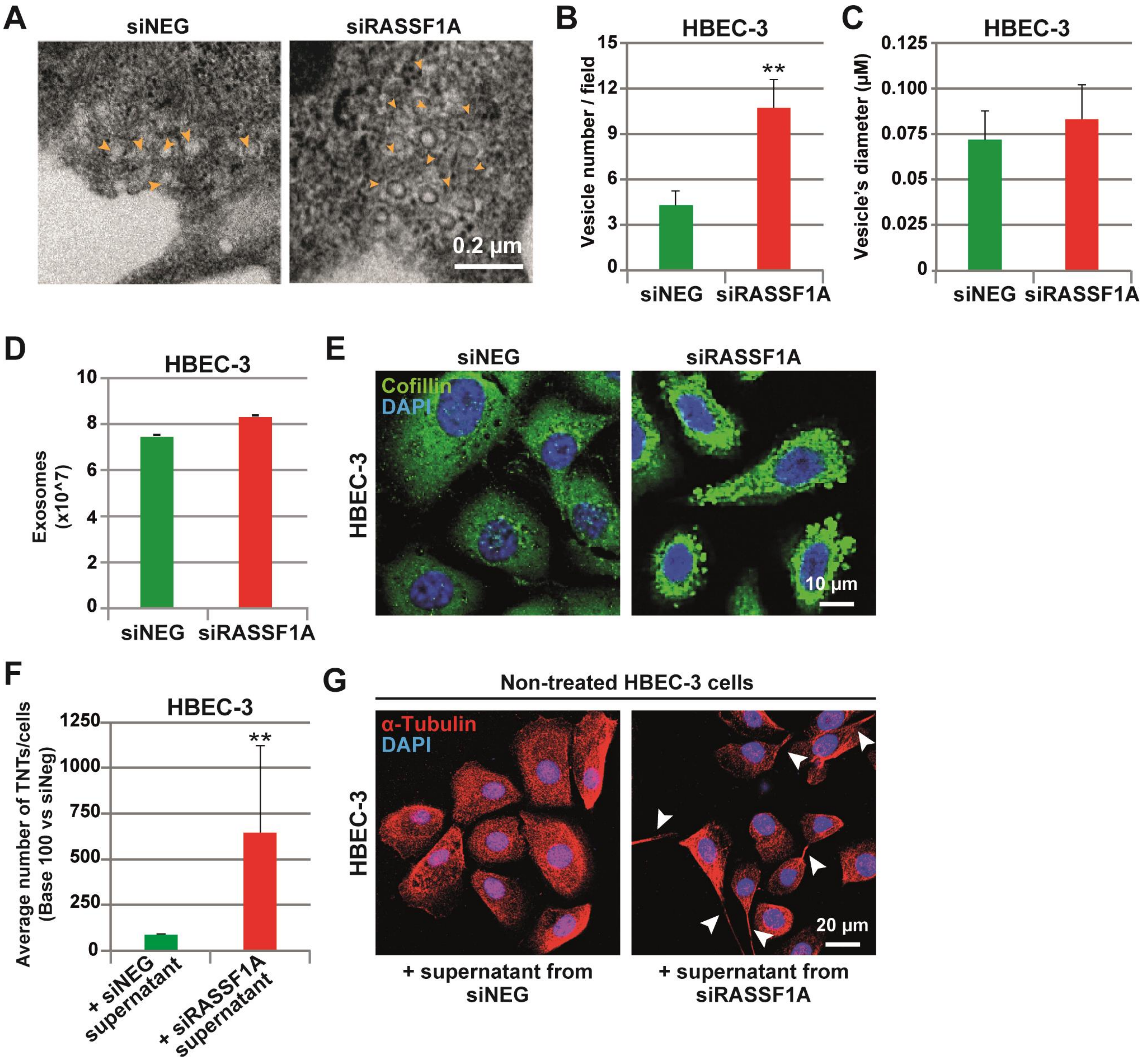

Figure 5: Exosomes released by RASS1A depleted cells affect TNTs formation.

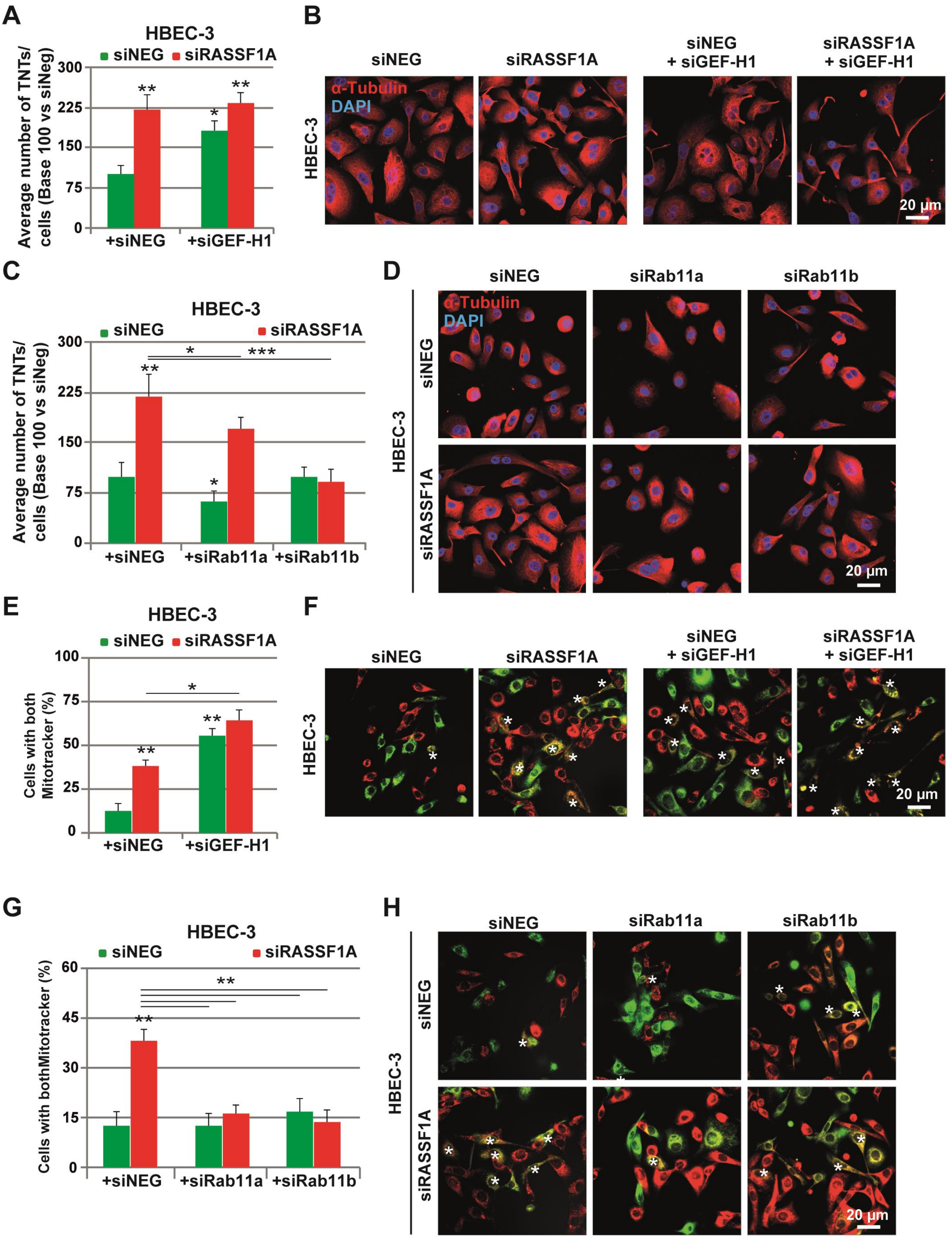

Figure 6 : TNTs formation in the absence of RASSF1A is dependent on GEF-H1 inactivation and Rab11 activation.

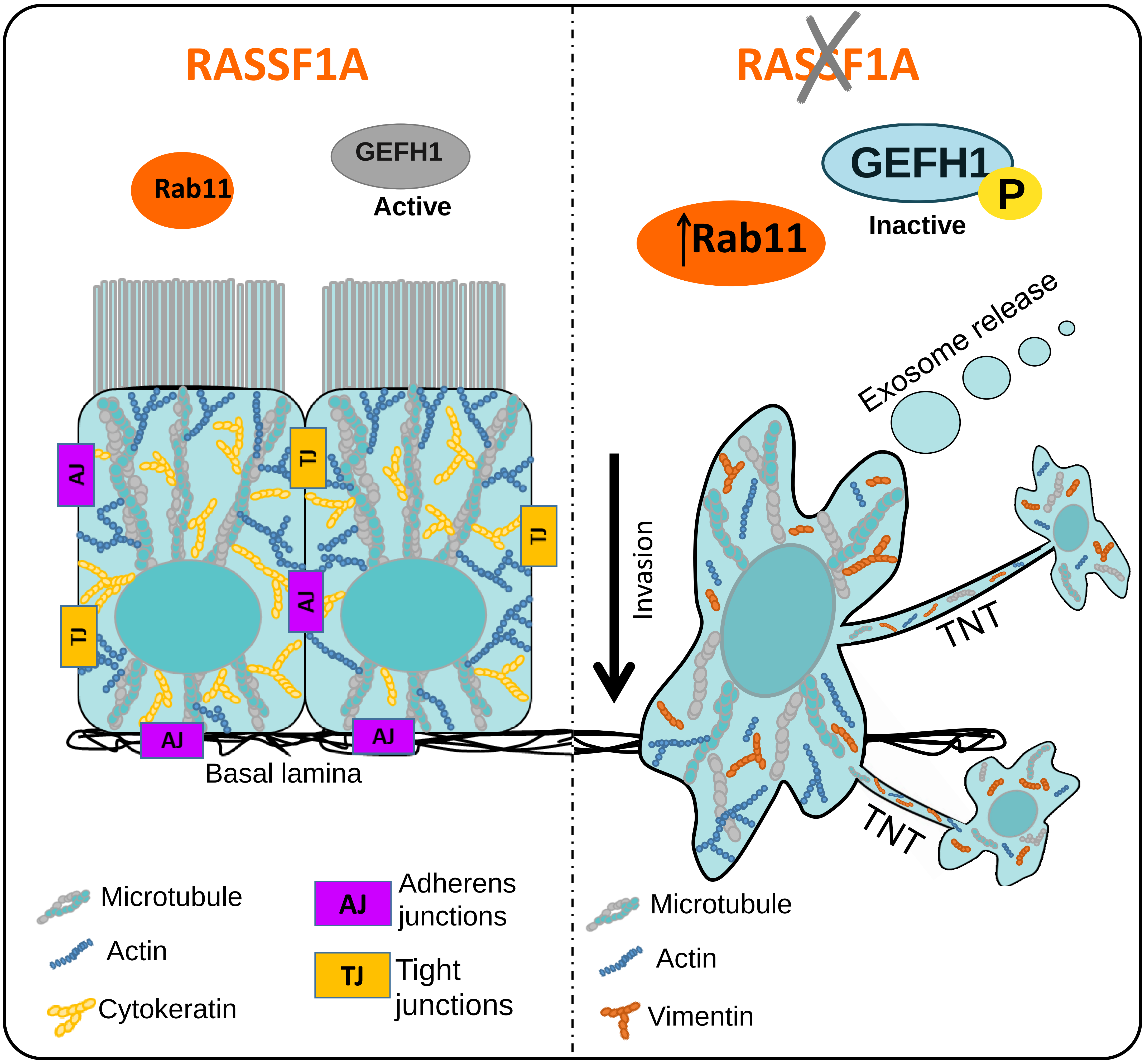

Figure7. RASSF1A prevents tunneling nanotube formation between cells through GEFH1/Rab11 pathway control.
