# Supplementary material for: A role for RASSF1A in tunneling nanotube formation between cells through GEFH1/Rab11 pathway control
Source: Cell Commun Signal. 2018 Oct 11;16:66. doi: 10.1186/s12964-018-0276-4 (PMC6180646; doi:10.1186/s12964-018-0276-4)
Supplement: Supplementary file 7 — Table S1. Characteristics of the cell lines used in the study. (DOCX 20 kb) [file 12964_2018_276_MOESM6_ESM.docx]

**Table S1 - Characteristics of the cell lines used in the study**.

| Origin | Cell lines | RASSF1A promoter gene | Gene Alteration Profiles |
| --- | --- | --- | --- |
| Bronchial epithelial cells | HBEC-3 | Unmethylated | Rb/p16 block |
|  | HBEC-3 RasV12 | Unmethylated | Rb/p16 block  Activating mutation of Ras |
|  | BEAS-2B | Unmethylated | Inactivating mutation of PP2A |
|  | BEAS-2B RasV12 | Unmethylated | Inactivating mutation of PP2A  Activating mutation of Ras |
|  | H1975 | Unmethylated | Activating mutation of EGFR  p53 mutation R273H  PI3K mutation  CDKN2A deletion  Deletion of p16 |
|  | A549 | Methylated | Activating mutation of RasS12  CDKN2A deletion  Deletion of p16 and p14  LkB1/STK11 mutation |
|  | H441 | Methylated | Inactivating mutation of p53  Activating mutation of Ras |
|  | H23 | Methylated | p53 mutation I246M |
|  | H1650 | Methylated | Activating mutation of EGFR  Inactivating mutation of p53  CDKN2A deletion  Deletion of p16 |
| Pleural mesothelial  cells | H28 | Methylated | Deletion of p16 |
|  | H2052 | Methylated | Deletion of p16 |
|  | H2452 | Unmethylated | Deletion of p16 |
|  | MSTO-211H | Unmethylated | Deletion of p16  Inactivating mutation of LATS1/2 |
